# Ecological and Biotechnological Aspects of Pigmented Microbes: A Way Forward in Development of Food and Pharmaceutical Grade Pigments

**DOI:** 10.3390/microorganisms9030637

**Published:** 2021-03-18

**Authors:** Ramesh Chatragadda, Laurent Dufossé

**Affiliations:** 1 Biological Oceanography Division (BOD), Council of Scientific and Industrial Research-National Institute of Oceanography (CSIR-NIO), Dona Paula 403004, Goa, India; 2 Chemistry and Biotechnology of Natural Products (CHEMBIOPRO Lab), Ecole Supérieure d’Ingénieurs Réunion Océan Indien (ESIROI), Département Agroalimentaire, Université de La Réunion, F-97744 Saint-Denis, France

**Keywords:** pigments evolution, biological properties, horizontal gene transfer, fluorescent pigments

## Abstract

Microbial pigments play multiple roles in the ecosystem construction, survival, and fitness of all kinds of organisms. Considerably, microbial (bacteria, fungi, yeast, and microalgae) pigments offer a wide array of food, drug, colorants, dyes, and imaging applications. In contrast to the natural pigments from microbes, synthetic colorants are widely used due to high production, high intensity, and low cost. Nevertheless, natural pigments are gaining more demand over synthetic pigments as synthetic pigments have demonstrated side effects on human health. Therefore, research on microbial pigments needs to be extended, explored, and exploited to find potential industrial applications. In this review, the evolutionary aspects, the spatial significance of important pigments, biomedical applications, research gaps, and future perspectives are detailed briefly. The pathogenic nature of some pigmented bacteria is also detailed for awareness and safe handling. In addition, pigments from macro-organisms are also discussed in some sections for comparison with microbes.

## 1. Introduction

The survival of life forms on earth is dependent on various pigments, including light-harvesting pigments like chlorophylls, phycoerythrin, and phycobiliproteins [1,2]; harmful light-filtering pigments like proteorhodopsins [3,4], melanin’s, pyomelanin, pyocyanin, fluorescent proteins; predator defending pigments like aplysioviolin [5], cephalopods ink [6,7], Dendrobatidae frog toxins [8], microbial pigments and so on [9]. The quantity, quality, and attractiveness of pigments from various sources such as microbes, algae, invertebrates, and macro-organisms may comprise either beneficial or toxic chemical constituents. Not all colors appealing to our eyes are beneficial to humans. Therefore, investigations on the chemistry of pigment molecules are gaining more interest in the current research. In 1666, Sir Isaac Newton had initiated the beginning of research on colors by developing the first circular diagram of colors, and later various researchers like Harris (1776) and Goethe (1810). Sir Humphry Davy demonstrated the causes of various colors of organic molecules [10]. Later in 1820, Friedrich Accum revealed the many side effects of synthetic colorants in various foods [11]. Sir William Henry Perkin was the first man to develop the first synthetic textile color compound “mauvine” in 1856. With this brief historical background, the visible spectral pigments and invisible nonspectral pigments gain more attention due to numerous applications in ecology, evolution, biomedical, and industrial perspectives. The international color symbolism chart indicates that each color has a specific meaning in different countries and cultures. Despite numerous known applications, evidence shows that visual pigments (color and light) can directly influence the brain [12], psychology [13], taste and flavor of humans [14,15,16], and science communication [17]. The lack of dietary pigments like carotenoids in our daily food intake may lead to various diseases and in rare case death [18]. Visual and food colorants are playing a significant role in decision making in our life to choose different foods and many other things [19], through vision, flavor, olfaction, gustation, and oral somatosensation ways [16].

Humans cannot see nonspectral colors due to a lack of trichromatic or tetrachromatic color vision-related cone types in their eyes. A recent study demonstrated humming birds’ ability to perceive nonspectral colors via the tetrachromacy phenomenon [20]; another example of categorical color perception was observed in Estrildid finches [21]. Numerous studies have been exploring the spectral pigments from microbes and higher organisms for various applications. Nevertheless, nonspectral pigments and their ecological importance in nature and biotechnological applications are not well studied. Thus, studies on nonspectral pigments remain a research gap in the current global science development scenario. Indeed, the planet earth is structured with visible and invisible micro and macromolecules produced by prokaryotes and eukaryotes, regulating various physical, chemical, biological, and geological processes. After going through a vast literature on microbial pigments, it is now understood that microbes and macro-organisms produce varied pigment molecules with a specific purpose in the respective milieus.

The resource of pigments, production rate, transport, price, sustainability, palatability, durability, effectiveness, legislative and regulatory approval, and demand by consumers are the primary requisites for various biotechnological applications in commercial industries. In this context, microbial pigments are attracting great demand to develop food grade, textile grade, and drug grade natural pigments. The reasons for high demand for microbial pigments are their promising unlimited resources, high production of required quantity of pigments, least cost-effective, easy cultivation and can be harvested throughout the year, adaptability to various environments, optimization, stability, genetic engineering, no side effects, eco-friendly, biodegradable, and indispensable applications in multidisciplinary aspects such as ecological, evolutionary, biomedical, agriculture, and industrial studies [9,22,23,24]. Many microbes are known to produce a wide variety of pigment molecules with innumerable biological properties and other industrial applications [9,25,26]. Especially, natural pigments of microbial origin have many advantages over synthetic pigments. Although artificial colors are more attractive and have been widely used around the world market (42%) [19,27,28,29,30], they are found to have many side effects (e.g., teratogenic, cancer, etc.) [29,30,31], and some are not biodegradable (e.g., textile dyes), causing health disorders to aquatic organisms and humans [32,33,34]. Hence, researchers are trying to find alternative physical, chemical, and biological methods to degrade synthetic colors [35,36,37] to avoid the side effects posed to the public and environmental health. Therefore, instead of developing synthetic colors and finding new methods for their degradation, exploring natural pigments from microbes would bring about innumerable advantages for the public and the environment.

Lack of knowledge on pigmented microbial isolation sources and their bioprospecting methods would make researchers face trials in microbial pigment research. Thanks must be extended to all the past researchers who explored the pigmented microbes from various environments and demonstrated numerous applications through various methods. Based on the published review of the literature [9,23,25,38,39,40,41,42,43,44,45], current researchers are looking for novel strains, new extraction techniques, and new applications of pigments. In this context, this review is intended to provide the current knowledge on various aspects of microbial pigments such as classification, evolution, horizontal gene transfer, market demand, spatial distribution, pigment therapy, and future perspectives.

## 2. Classification of Pigments

Microbes display all kinds of color hues such as black, blue, bronze, brown, cream, grey, green, orange, purple, indigo, pink, red, yellow, metallic green, red, yellow, and rainbow. These pigments can be classified into various categories based on their visual, chemical, and spectral properties and source of origin (based on mobile genes) [9]. Based on visual appearance, prokaryotes and eukaryotes display monochromatic to polychromatic pigment combinations within the Munsell color system. Some higher organisms like dragonfish [46,47] and hummingbirds [20] exceptionally display or see colors beyond our visible spectrum and near-infrared spectrum. These incidents suggest that humans lack nonspectral cones to perceive colors existing beyond the visible spectrum. Visually, pigments represent the following phenomena on earth: (1) Natural pigments, (2) Bioluminescence, (3) Fluorescence, and (4) Iridescence (structural colors), and (5) Non-spectral colors. Humans can perceive all the color phenomena except non-spectral colors.

**Figure 1:**
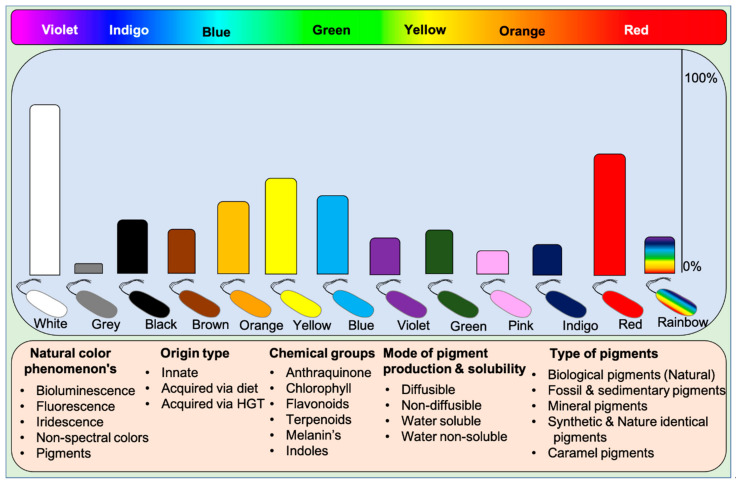
A wide array of pigmented microbes seen in nature. The abundance of the type of pigmented bacteria is depicted in bars based on the available literature. Rainbow bacteria are iridescent. Classification of pigments based on various aspects of biochromes. Chlorophyll pigments are not included in the data as they are ubiquitous. HGT: Horizontal gene transfer.

Functionally, five different types of pigments are found in nature: (1) Biological pigments, (2) Fossil and sedimentary pigments, (3) Mineral pigments, (4) Synthetic & identical natural pigments, and (5) Caramel pigments (Figure 1). Biological pigments are derived from live microbes, plants, and animals. In contrast, fossil pigments are indeed biologically originated but preserved in fossils for millions of years, acting as evolutionary evidence [48,49,50,51,52,53]. In rare cases, fossil pigments can be of synthetic origin [54]. Mineral pigments are inorganic insoluble pigments used in artistic, cosmetic, archeological, and evolutionary studies [55,56,57,58,59,60]. In contrast, synthetic colorants are synthesized in the laboratory for food colorants and dyeing applications [61]. Dozens of synthetic colorants are being used in food and beverages [61,62]. Caramel pigments are natural sugar-based colorants used in a variety of food and beverage products. These caramel colors are classified into Caramel I, II, III, and IV classes to fulfill the requirement of food systems [63]. Solvatochromicity of these pigments varies according to the extraction solvent.

Based on chemical groups, microbial pigments are broadly differentiated into anthraquinones, carotenoids, indoles, phycobiliproteins, prodigiosin, rhodopsins, melanins, and violacein [9,64]. For understanding the evolutionary aspects, rhodopsins, melanins, and iridescent (structural) pigments are briefly discussed herein. Microbial rhodopsins are light-harvesting photoproteins that bind to retinal and respond to light, which has evolutionary importance. These rhodopsin are found in Archaea, bacteria, fungi, viruses [65], and some eukaryotes [66]. Based on the known functions, rhodopsins are classified as light sensors (rhodopsins, opsins), energy-conserving transmembrane proton pumps (bacteriorhodopsins, proteorhodopsins, and xanthorhodopsins), and transmembrane chloride pumps (halorhodopsins) [4]. In Haloarchaea, a single cell can possess multiple rhodopsins with varied functions [4]. Melanins are biosynthetically, functionally, and structurally diverse pigments, including five known groups of allomelanin, eumelanin, and neuromelanin pheomelanin, and pyomelanin [67]. It is often easy to isolate monochromatic pigment-producing microorganisms from different environments, but isolation of polychromatic pigments producing bacteria such as *Pseudomonas aeruginosa* (blue and green pigments), *Streptomyces* sp. (yellow, orange and brown) [25] and iridescent or shimmering bacteria (VIBGYOR) [68] (https://www.hoekmine.com; accessed on 10 January 2021; Hoekmine BV, 2020) are rarely isolated. Structural colors are also recorded in fossil feathers, suggesting the importance of evolutionary aspects [69].

In general, microbes possess innate pigment traits, but some non-pigmented microbes acquire pigment traits from pigmented microbes (see the Section below: Horizontal Gene Transfer). For this reason, microbial pigments are classified as innate pigments and acquired pigments. Often, pigmented microbes release diffusible and non-diffusible pigments in culture media. However, rarely, some pigments are water-insoluble, for instance, blue pigment indigoidine [70], red pigment [71], and violacein [72]. Some pigments even do not dissolve in solvents; in such incidents, resin extraction can be employed to extract pigments.

## 3. Functions of Microbial Pigments

**Figure 2:**
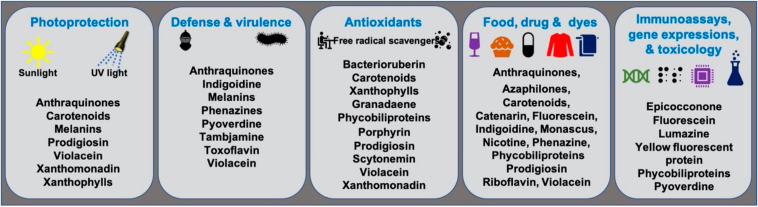
Ecological functions and other applications of important microbial pigments.

Microbial pigments are known to play a variety of ecological functions in their milieus. (Figure 2). Antioxidant properties of different microbial pigments are detailed in the supplementary file provided in the previous review published in 2019 (see supplementary file) [9]. Prodigiosin pigment produced by some strains of *Vibrio* sp. function as photoprotectants against UV light [73]. Violacein pigment of *Janthinobacterium lividum* and *Chromobacterium violaceum* demonstrated antipredator activity against bacterivorous nanoflagellates, indicating its defensive function [74]. *J. lividum* associated with the skins of some frogs and salamanders, secretes violacein pigment to protect them from pathogenic fungi, *Batrachochytrium dendrobatidis* [75,76,77]. Phenazine compounds produced by bacteria play multiple functions, including chemical signaling, biofilm formation, survival, and virulence [78]. Pyoverdine, a fluorescent yellow-green pigment, regulates iron transport and virulence functions in *Pseudomonas fluorescens* [79]. Tambjamine, a yellow pigment produced by *Pseudoalteromonas tunicata* [80], is suggested to help its host prevent other predatory fouling organisms [81]. Likewise, indigoidine, a blue pigment produced by *Phaeobacter* strains, is suggested to inhibit competing bacteria in the environment [82]. Bacterial melanin pigments act as photoprotectants [83,84,85,86,87]. For instance, *Vibrio cholerae* melanins serve as survival fitness factors when physico-chemical factors become unfavorable [88]. Some endophytic fungi releases anthraquinones, to protect the host plant from damage due to insects and microbes [89]; while, fungal melanins demonstrate multiple functions [90].

Bacteriochlorophylls are photosensitizers (light harvesters) in photosynthetic bacteria but absent in non-photosynthetic bacteria [91]. Non-photosynthetic bacteria may utilize a self-photosensitization mechanism [92]. In photosynthetic and non-photosynthetic bacteria, carotenoids, the accessory photosynthetic pigments act as photoprotectants and antioxidants, thus protecting cells from damage due to UV and sunlight illumination [91,93,94]. Bacterial communities in the air-water interface did produce more pigmentation to tolerate sunlight and are relatively drug-resistant compared to non-pigmented bacteria [95]. The extremophilic bacteria isolated from salt lakes [96] and cold environments like Antarctica [97,98] adopt environmental stress with carotenoids and other pigments. The yellow pigment of *Thermus* was proposed as a photoprotectant [99]. Carotenoids of archaea [100], yeasts [101,102], cyanobacteria, and algae [103] also function as photoprotectants. Marennine, a blue pigment produced by diatom *Haslea* is involved in greening on oysters [104], and displayed a prophylactic effect [105,106]. Food colorants, drug, dye, and other biotechnological applications of microbial pigments are detailed in the section below.

## 4. Pathogenicity of Pigmented Microbes

Despite the numerous known pigments’ applications, the literature suggests that some pigmented bacteria are emerging as pathogens in aquaculture farms and even in humans. Violacein-producing bacterium *Chromobacterium violaceum* has been reported to cause infections in children and adults [107]. *Janthinobacterium lividum*, another violacein-producing bacterium, resulted in mass mortality of rainbow trout *Oncorhynchus mykiss* in the hatchery from Korea [108]. Prodigiosin producing *Serratia marcescens* also infects insects, other invertebrates, and vertebrates, including humans [109,110]. Strains of *S. marcescens* and *C. violaceum* are reported to be opportunistic pathogens to humans [111,112]. In all these cases, there is no evidences about the role of violacein and prodigiosin pigments in virulence function. A recent study demonstrated that prodigiosin pigment did not play a virulence function in entomopathogenic *S*. *marcescens* [113].

However, few pigments such as bacterial melanins [114] and pyoverdines [115] regulate virulence function. The red pigment producing fungi such as *Fusarium* and *Monascus* produce mycotoxins (e.g., citrinin and 4,15-diacetoxyscirpenol) linked to pathogenicity [116]. Thus, researchers are searching for fungal species that do not produce any toxins [117]. We suggest that determining an isolated pigmented microbe’s pathogenicity (hemolytic activity) would help to avoid infections and mortality.

## 5. Horizontal Gene Transfer (HGT) of Pigment Genes

In the last two decades, studies observed rare incidences of acquisition or transfer of pigment genes between related and non-related microbial communities. The transfer or acquisition of pigment genes between various micro-organisms is a sign of environmental function. The acquired pigment trait acts as a defensive mechanism against other microorganisms, acting as sunscreen (photoprotection) against UV rays and harvests light for enhanced photosynthesis. This is an exciting area of research to study the ecological importance of pigment gene transfer among microbes.

**Figure 3:**
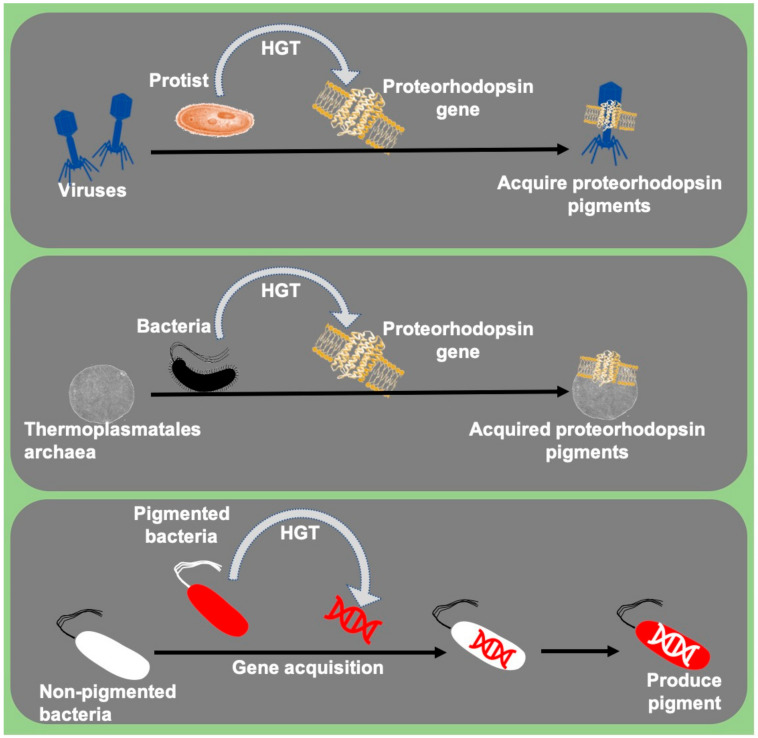
Acquisition of pigment encoding genes by Archaea, bacteria, and viruses.

Genes coding for light-harvesting pigment proteins such as proteorhodopsins were reportedly transferred between planktonic bacteria and archaeal communities dispersed only in the photic zone [4]. These proteorhodopsins encoding genes reportedly acquired by eukaryotes, dinoflagellate protists from bacteria [66], and protists’ viruses [65]. Bacteria like *Collimonas* CT were suggested to produce blue pigment (violacein) via pigment gene acquisition, probably acquired from *J. lividum* and/or *Duganella* sp. [118] (Figure 3). *LuxA* genes responsible for light emission in the luminescent bacteria were also reportedly acquired by non-luminous vibrios through HGT and become luminescent [119]. Similarly, pathogenicity-related genes were also shared among many bacteria via HGT [120]. Studying the HGT mechanisms in these microbes will help us to understand the role of HGT in evolution.

## 6. Cosmopolitan Distribution of Pigmented Microbes

**Figure 4:**
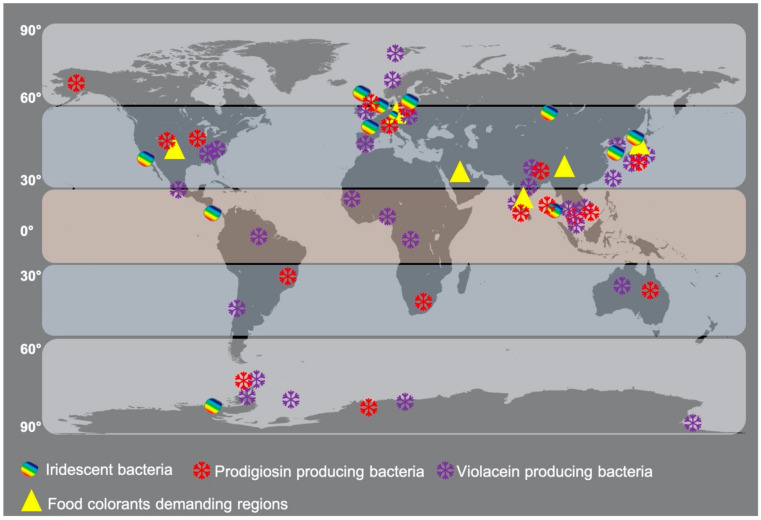
Cosmopolitan distribution of well-known pigmented microbes in different geographical areas.

The distribution patterns of well-known pigmented microbes have not been detailed in the literature to understand their evolutionary spread in different geographical environments. The current literature published so far reveals that pigments are environment-specific, depth-specific, host-specific, and functionally distinct [9,121]. Chlorophyll pigments are ubiquitous, whereas other pigment molecules are not widespread but restricted to specific groups of bacteria, indicating the evolutionary importance of pigments. To link the evolutionary concept with microbial pigment distribution, the well-known prodigiosin, violacein, and iridescent bacteria are mapped in this review (Figure 4). The map shows the cosmopolitan distribution of these bacteria in tropical, subtropical, and temperate environments. This spread pattern will help us to understand the hydrothermal vent-based origin of life theory by testing presence and absence, abundance, and low levels of microbial pigments in coastal and deep-sea environments of different geographical areas. Thus, further in-depth studies are required to link their distribution patterns to evolutionary studies.

## 7. Evolution of Pigments

From the evolutionary perspective, the origin of microbial pigments remains very little known. It is well understood that all the chemical molecules have originated from the origin of elements process [122]. Pigments of prokaryotes and eukaryotes display specific ecological and bioactive functions [9,123,124]. Pigments are also identified in non-living matters like fossils, sediments, and inorganic minerals [49,125]. Fossil pigments [49,126] and sedimentary pigments [125,127] are gaining in ecological and evolutionary importance to study environmental and population dynamics and chemical constituents of the past. Microbial pigments are ubiquitous in different environments at various depths and evolved for a specific function in respective milieus [9]. In contrast to microbial pigments, mineral pigments are intensely colored inorganic molecules with potential applications in artistic, cosmetic, forensic, archaeological, and evolutionary perspectives [55]. In the evolutionary perspective and according to the clay-mineral theory on the chemical origin of life [128] and recent evidences [129], we may be able to interlink the origin of molecules, including pigments in protocell, which helped protocells to survive in extreme conditions and supported the formation of multicellular organisms.

Since protocells’ origin, natural pigments have transformed into various phenomena such as pigments, fluorescence, and bioluminescence, found in prokaryotes and eukaryotes. Currently, researchers believe that life had originated 4.5 billion years ago from the extreme environment like hydrothermal vents in the ocean [130] or warm water pools in the volcanic land or geothermal (hot spring) areas [129,131,132], based on the evidences of hypothetic protocell structures, i.e., vesicles formed by simple fatty acids [130] and proteins [133], RNA [131] and DNA molecules [129]. The abundant external red pigments seen in deep-sea tubeworms at hydrothermal vents are indeed hemoglobins that act as binding sites to oxygen and hydrogen sulfide and transport these molecules to internal bacterial symbionts [134]. In contrast, the evidence of opsins and pigment molecules in thermal vents is not as abundant as in the photic zone or terrestrial environments. Deep-sea microbial pigments is underexplored due to difficulties in the culture and maintenance of samples under in situ conditions. Opsins are phylogenetically well-diversified and structurally different light sensors observed in prokaryotes [135,136], invertebrates, and vertebrates [137,138]. Opsins sense light and respond to physiological, chemical, and behavioral functions, and develop evolutionary adaptations. Phycobiliproteins are light-harvesting chromophores present in cyanobacteria and some algae, whose evolutionary origin is related to globin proteins and GC contents [139]. Efforts to understand the evolution of phycobiliproteins in cyanobacteria [140,141] and algae [139,142] using specific genes and targeted molecules is underway. Light-harvesting pigments, phycobiliproteins, and chlorophylls might have arisen independently several times in different lineages [143]. A piece of evidence exists on the origin and biosynthesis of bacteriochlorophyll *a* by a bacterial enzyme “3-vinyl-bacteriochlorophyll hydratase [144], suggesting the origin of enzymes first, followed by notions of coexistence of RNA and DNA [145] or homogenous RNA world [131,146,147] or DNA world [148], or still debating prebiotic DNA world [149]. The origin of other microbial pigments (e.g., prodigiosin, violacein, etc.) also needs to be evaluated for in-depth understanding and to interlink the evidence.

The chemistry and mechanisms involved in forming pigments in protocells and their divergence into different lineages are yet to be unveiled. The lack of enough evidence of protocells in the environment makes it difficult for researchers to understand protocells’ exact origin. The exact environmental conditions that favored protocells to develop various pigments are unknown. These pigments might have evolved to tolerate the intense illumination during the early earth formation generated from the chromosphere, photosphere, and atmosphere. This research angle remains untouched concerning the evolution of chromophores. Further detailed investigations on spatial and temporal patterns of various pigmented microbes from different environments and their complete genomics, proteomics, and chemicalomics may reveal some clues on the origin, evolution, and inheritance of pigments from protocell to eukaryotes. A recent conceptual study provides a new idea to understand the synthesis and development of prebiotic molecules in primitive cells [150]. Robotics based chemical synthesis studies have been arising in recent times [150,151], which may help us to understand the possible ways of origin of primitive molecules. However, this concept is still to be validated in real time, based on field evidences rather than empirical evidences. In the coming two to three decades, life’s true origin is expected to be in complete light with integrated evidence.

## 8. Pigment Gene Cassettes

Microbes producing high pigment yield are the primary research targets for commercial purposes. Many natural microbes have failed to produce the expected yield of pigments for food, drug, cosmetics, and textile applications. Therefore, exploring the entire pigment gene cassette of an interested microbial species is found to be the best approach to achieve high pigment yield through recombinant DNA technology. Some researchers might not be aware of the genes responsible for pigments; thus, this section has garnered information on different microbes’ gene clusters. *Pig* gene cluster for prodigiosin biosynthesis in *Serratia marcescens* [152,153], and *red* gene cluster for undecylprodigiosin biosynthesis in *Str. coelicolor* A3(2) were identified [154]. Prodigiosin synthesizing genes in *Hahella chejuensis* KCTC 2396, and *Pseudoalteromonas* species were identified as *hap* gene cluster [155]. Indigoidine biosynthesizing gene cluster in *Phaeobacter* sp. strain Y4I encoded as *igi* operon [82]. Violacein biosynthetic gene cluster “*vio*” was identified in *Chromobacterium violaceum* [156] and *Pseudoalteromonas* species [157]. Tambjamine, a yellow pigment of *Pseudoalteromonas tunicata* is synthesized by *tam* gene cluster [158]. Pyomelanin synthesizing genes were named as *hatABCDE* operon [159]. Bikaverin, a reddish pigment produced by *Fusarium fujikuroi,* carries bikaverin synthesizing *bik* gene cluster [160]. *Monascus* red pigments biosynthesizing genes in *Monascus ruber* and *M. pilosus,* are designated as *MrPig,* and *mok* gene clusters, correspondingly [161,162]. Other strains of *M. pilosus* possess *MpPKS5* and *mpp* genes [163], whereas *M. purpurea* bears *MpPKS9* and *mok* gene cluster [164]. The *crt* genes are involved in the biosynthesis of carotenoids in *Brevundimonas* sp. [165], *Hematococcus pluvialis* [166], *Deinococcus wulumuqiensis* [167], *Xanthophyllomyces dendrorhous* (*Phaffia rhodozyma*) [168], Antarctic bacteria [169], and other bacteria [170]. *Dunaliella* sp. carotenoids are mainly triggered by two essential genes *CGP* and *LCYB* [171]. In *Rhodotorula mucilaginosa*, *CAR* gene cluster synthesizes carotenoids [172].

## 9. Substrates, Mutagen Agents, and Adsorbents

The use of natural agro-industrial wastes has been a recent trend and strategy in the biotechnological process to increase pigment yield. The natural and genetically engineered microbes are subjected to fermentation studies to identify the optimal culture conditions for maximum pigment yield with various substrates (Table 1). A variety of cost-effective substrates such as copra seed, peanut seed, sesame seed, coconut oil, peanut oil, sesame oil [173], sunflower oil [174], peanut powder [175], corn steep liquor, cassava waste [176], squid pen powder [175], brown sugar [177], tannery fleshing [178], ram horn peptone [179], kitchen waste [180], wheat bran [181], casein, sweet potato powder [182], bagasse [183], saw dust, palm oil fiber and rice husk [184] had been utilized to enhance and improve the prodigiosin pigment production from *S. marcescens*. Violacein production rate was increased using brown sugar, molasses, sugarcane bagasse, and pineapple waste [185,186]. The enhanced production of pyocyanin from *Pseudomonas aeruginosa* was successful with cottonseed meal [187]. 

*Monascus* pigment production was enhanced by utilizing tapioca starch [188], cassava powder, coconut oil cake, groundnut oil cake, jackfruit seed powder, rice bran, palm kernel cake, sesame oil cake, spent brewing grain, tamarind seed powder, wheat bran [189,190], cheese whey, grape waste, rice hulls, soybean bran [191], coconut residue, cornmeal, peanut meal, soybean meal [192], corn cob [193], jackfruit seed [194], a variety of rice [195,196,197], durian seed [198], sugarcane bagasse [199], sweet potato [200], and brewery’s spent grain [201]. 

Carotenoids production in yeasts was improved by supplementing peat extracts [202], grape juice [203], beet molasses, glucose syrup, grape must, maize flour extract, soybean flour extract [204], cane molasses [205,206,207], sugar cane juice [208], corn syrup [207,209], coconut milk [210], brewer malt waste [211], corn meal [212], mustard waste [213], raw malt extract [207], tomato waste [214], chicken feather peptone [215], whey filtrate, coconut water [216], date palm waste, maize waste, mango peels, onion waste, peanut leaf and fruit wastes, potato peels, rice straw, sugarcane waste, wheat straw [217], and powders of onion peel, mung bean, pea pods and potato skin [218].

**Table 1:** Substrates promoting high pigment yield from various microbes are alone detailed herein for further biotechnological applications.

Organism	Substrate	Pigment	Maximum Pigment Yield	Reference
**Bacteria**				
*S. marcescens*	Peanut seed broth	Prodigiosin	38.75 mg/mL	[173]
*S. marcescens*	Cassava waste	Prodigiosin	49.50 mg/mL	[176]
*S. marcescens*	Tannery fleshing	Prodigiosin	33 mg/mL	[178]
*S. marcescens*	Ram horn peptone	Prodigiosin	27.77 mg/mL	[179]
*S. marcescens*	Kitchen waste	Prodigiosin	22.3 mg/mL	[180]
*S. marcescens*	Bagasse	Prodigiosin	40.86 g kg^−1^	[183]
*S. marcescens*	Sunflower oil	Undecylprodigiosin	7.90 mg/mL	[174]
*Chromobacterium violaceum*	Liquid pineapple waste	Violacein	57.90 mg/mL	[185,186]
*Pseudomonas aeruginosa*	Cotton seed meal	Pyocyanin	9.2 μg/mL	[187]
**Fungi**				
*M. purpureus*	Jackfruit seed	Monascus	10.2 OD/g	[189]
*M. purpureus*	Grape waste	Monascus	20–22.5 g/L	[191]
*M. purpureus*	Corn meal	Monascus	129.63 U/g	[192]
*M. purpureus*	Corn cob	Monascus	25.42 OD/g	[193]
*M. purpureus*	Brewery’s spent grain	Monascus	16.75 UA_500_	[201]
**Yeast**				
*Rhodotorula rubra*	Peat extract	β-Carotene	1256 μg g^−1^	[202]
*Rhodotorula glutinis*	Grape must	Carotenoid	915.4 μg g^−1^	[204]
*Rh. glutinis*	Molasses	Carotenoid	185 mg L^−1^	[206]
*Rh. glutinis*	Chicken feather peptone	Carotenoid	92 mg L^−1^	[215]
*Xanthophyllomyces dendrorhous*	Grape juice	Astaxanthin	9.8 μg mL^−1^	[203]
*X. dendrorhous*	Mustard waste	Astaxanthin	25.8 mg L^−1^	[213]
*X. dendrorhous*	Molasses	Carotenoid	40 mg L^−1^	[205]
*X. dendrorhous*	Coconut milk	Astaxanthin	850 μg g^−1^	[210]

UA: Absorbance units; OD: Optical density.

Mutagenic agents such as various chemical reagents, UV illumination, and gamma radiation have been used to enhance pigment production from natural and recombinant microbial strains [219,220]. Carotenoid content of *Rhodopseudomonas palustris* was stimulated with blue, yellow, white, green, incandescent lamp, red, halogen, and fluorescence lamp [221]. The enhanced prodigiosin production was successful with gamma radiation [219]. Stimulated pigment production in filamentous fungi was evident with blue (for carotenoids) [222], green, red, and UV-light (red pigment bikaverin) [223]. Mutations in the genes caused *Fusarium fujikuroi* to produce different hues of pigments [160]. For yeasts, low energy ion beam implantation [224], gamma radiation [225], light-emitting diodes [226,227], and UV light [228] were used as an effective approach for carotenoids enhancement. High production of phycobiliproteins was achieved from cyanobacteria, *Pseudanabaena mucicola* cultures grown under white light [229]. The increase of phycocyanin production from *Spirulina platensis* [230] and *Pseudanabaena* sp. [231] was evident under red light. Maximum production of phycoerythrin and carotenoids from *Pseudanabaena* sp. was observed in green light [231]. In unicellular microalgae, carotenoids production is enhanced through UV radiation [232,233,234,235], blue and red light [236,237], light-emitting diodes [238,239], and various toxic chemicals [233,240].

The use of various adsorbents in microbial fermentation appears to be the most effective strategy to enhance pigment production and maximum pigment recovery. Studies have utilized different internal adsorbents for maximum pigment recovery. Treating culture flasks with Sigmacote to reduce attachment of pigment cells to a glass surface [241]; use of resins like X-5, HZ806, and HZ802 in cultures for pigment adsorption [242]; adding rice husks [243] or alginate beads to cultures for adsorbing more pigment cells [244]; addition of Diaion HP-20 resin [245,246,247] and polyurethane foam cubes [248] to cell culture are the additional strategies in prodigiosin pigment recovery. Monascin pigments are recovered by adding rice, called “red mold rice” [161]. High monascus pigment yield was achieved with stirred drum bioreactor [249]. Various extraction techniques such as ionic liquid–assisted extraction, microwave-assisted extraction, ultrasound-assisted extraction, pressurized liquid extraction, pulsed electric field assisted extraction, and supercritical CO_2_ extraction are employed to recover pigments from fungi [220]. Finding the new adsorbents and extraction techniques to recover pigments are important requisites in microbial pigment research.

## 10. Biomedical and Industrial Applications

**Figure 5:**
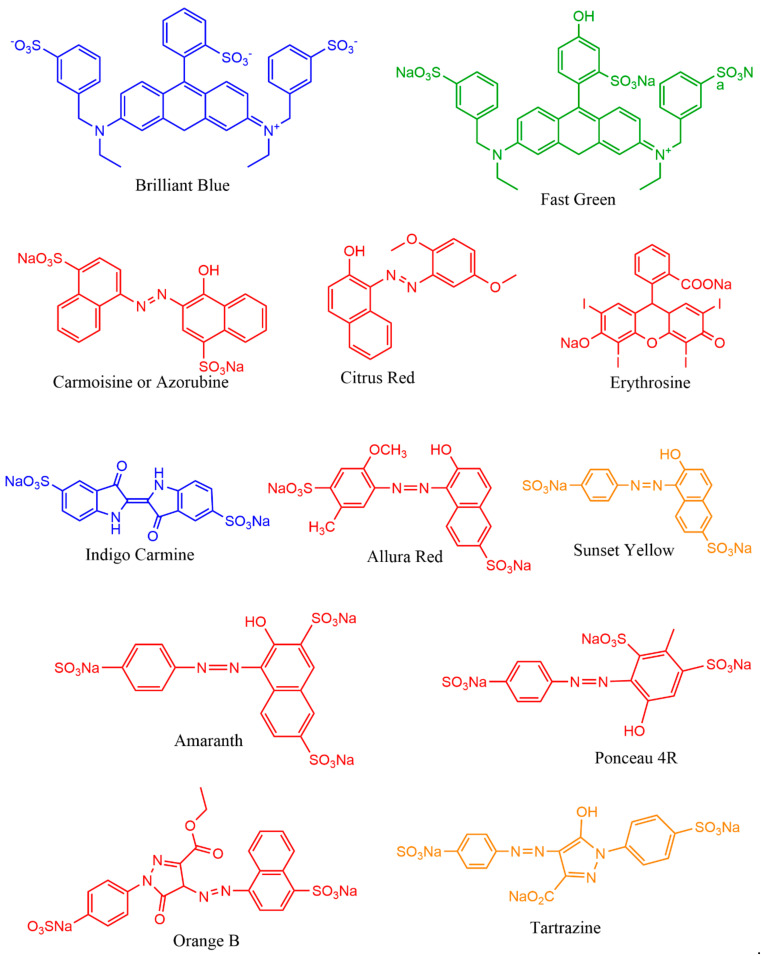
Chemicals structures of synthetic pigments.

**Figure 6:**
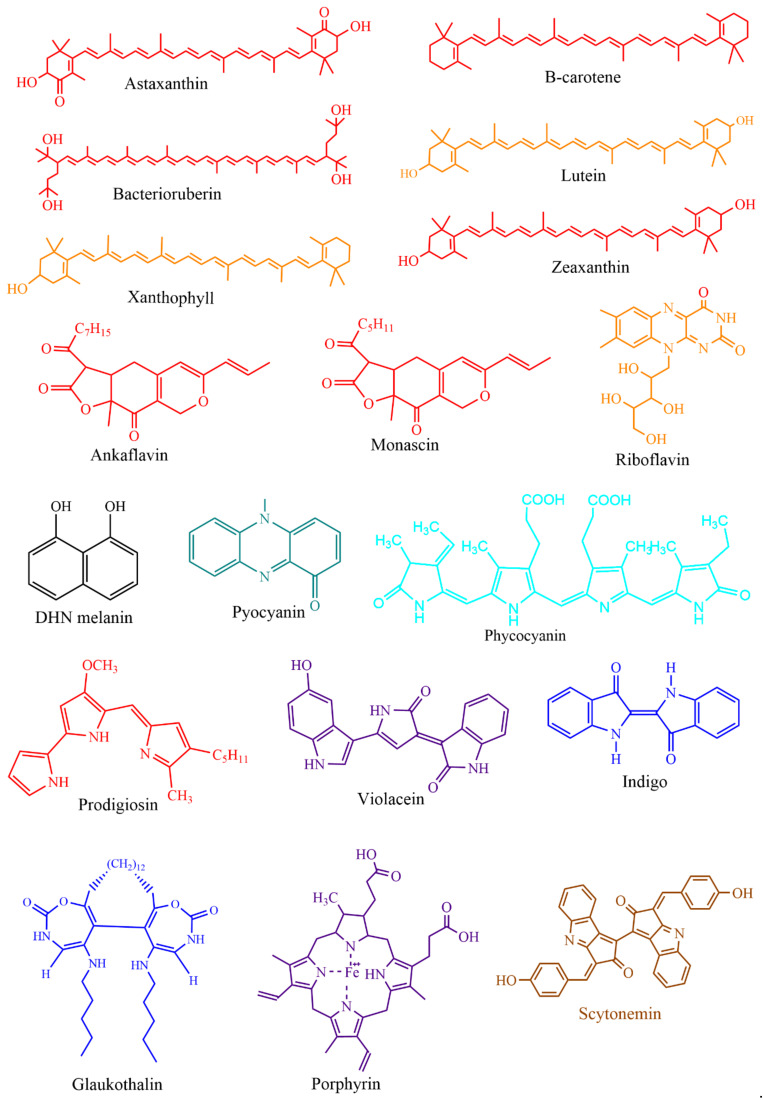
Chemical structures of important microbial pigments.

This section provides various applications of microbial pigments that were not covered in the previous review [9]. Dozens of synthetic and natural pigments have been used in beverages, foods, dyeing, and textiles (Figure 5 and Figure 6). A red-pigmented (related to carotenoid) *Arthrobacter* sp. offer the antitumor activity against esophageal cancer cells [71]. Prodigiosin produced by *Pseudomonas rubra* displayed antimicrobial activity against pathogenic bacteria and yeast [250]. Prodigiosin extracted from *S. marcescens* displayed potential insecticidal activity against *Drosophila melanogaster* larvae [175], ants, cockroaches, and termites [251]. Prodigiosin and glycolipid biosurfactant’s synergistic effect demonstrated antimicrobial activity against pathogenic bacteria [252]. Prodigiosin extracted from *Zooshikella* sp. and *Streptomyces* sp. and other pigments from marine bacteria displayed potential application in staining and food colorants [253]. Currently, in our lab, calcium oxalate and uric acid stones dissolving pigments from marine bacteria are being isolated. Prodigiosin from *S. marcescens* [254] and violacein from *C. violaceum* [255] promise to treat the chagas disease. Violacein pigment is employed in cotton fabrics dyeing [256], and lead detecting whole-cell lead biosensor [257]. Violacein produced by *Microbulbifer* sp. demonstrated antinematode activity against *Caenorhabditis elegans* [258]; a strain of violacein producing *Chromobacterium* isolated from the Himalaya region, produced by bioplastic polyhydroxyalkanoates [259].

Indigo pigment isolated from *Pseudomonas* sp. displayed antioxidant property [260]. Glaukothalin, a blue pigment produced by *Rheinheimera* sp., showed antibacterial activity against few marine bacteria [261]. Pyocyanin from *Pseudomonas aeruginosa* demonstrated textile dyeing properties, antifungal activity against blast fungus, *Magnaporthe grisea,* and antibacterial properties against blight of rice, *Xanthomonas oryzae* [262]. Micrococcus sp.’s yellow pigment showed excellent wound healing and anti-inflammatory property in albino rats [263]. Bacterioruberin carotenoids of halophilic bacteria have significant antioxidant and antibacterial activities [264]. Microbial pigments (Actinorhodin, carotenoids, flexirubin, melanin, phycocyanin, phycoerythrin, blue pigment) are also used to synthesize various nanoparticles with biological properties like antioxidant, antimicrobial, anticancer activities [265].

Fungal pigments were reviewed to have a wide range of applications in food colorants [266,267], bioactive properties, and textile dyeing [40,268,269,270]. In contrast, bacterial pigments like prodigiosin and violacein are used to color papers, candles, soaps, ink, clothes [271], and textile dyeing [272]. Monascus pigment or anthocyanin pigment are employed as noninvasive dye indicators in safe cell viability assay for *Paramecium* [273], *Euglena* [274], and breast cancer cells [275]. Microbes isolated from cryosphere environments also produced various pigments with multifaceted applications [269,276,277], including anticancer activities [278].

Carotenoids of archaea [100] and Thraustochytrids [279,280] have potential nutraceutical applications. However, pigments from marine archaea and protists remain the least studied groups. Red algae extracts are used to make L’Oreal Paris Pure Clay Mask for skin glow and smoothening. Similarly, other commercial cosmetic products have been developed from cyanobacteria and microalgae [281]. Phycobiliproteins from cyanobacteria and algae demonstrated cosmetic, dye, nutraceutical, and bioactive applications [282,283,284]. Marennine, a blue pigment produced by diatoms, *Haslea* species, promises antimicrobial, antiviral, anticancer, and antioxidant activities [285].

## 11. Photo-Pigment Therapy

The combinations of light and pigments were found to be an effective strategy in antimicrobial assays. A study found that the bactericidal effect of blue light irradiated intracellular black pigment (protoporphyrin IX) on *Porphyromonas gingivalis* [286]. Likewise, flavin mononucleotide activated by blue light resulted in inhibition of *Staphylococcus aureus* biofilm [287]. Such strategies may be adopted and employed to increase the bioactive effectivity of microbial pigments against various pathogens. 

## 12. Market Demand for Microbial Pigments

**Figure 7:**
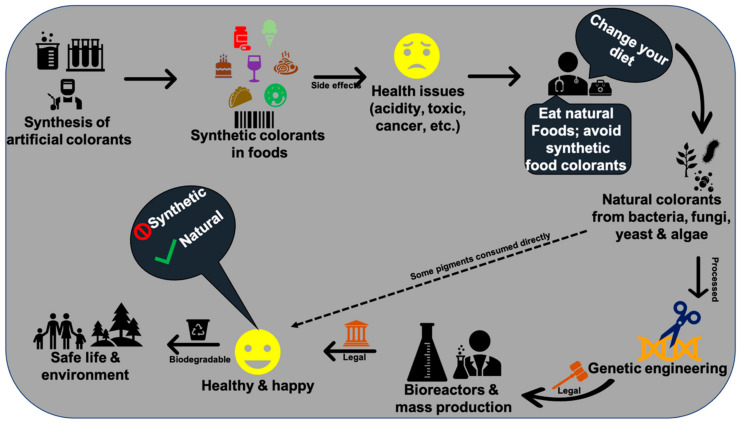
An illustration explaining the requirement of natural colorants over synthetic colorants.

In recent times, people around the world have come to know the harmful effects of synthetic colorants in foods (Figure 7). Thus, demand on natural pigments is increasing over artificial colorants. In 1971, the United States spent around 1 billion US dollars to increase the supply of natural colorants from various natural resources [288]. There are inadequate or scarce data on the global market value of food-grade microbial pigments. Very few pigments such as β-carotene, astaxanthin, and monascus are available in the market. Lack of surveys and literature on microbial pigments’ cost and demand are becoming hurdles to estimate the actual global market demand on microbial pigments.

Monascus pigments are traditional food colorants widely used in southeast Asian countries, which had an estimated market value of $12.0 million dollars during 1992 [289]. Monascus pigments are prohibited in the United States and Europe due to the presence of mycotoxins [290]. The global commercial market value for carotenoids reached $1.2 billion in 2010, $1.5 billion in 2014, and is expected to reach $2.0 billion by 2022 [228,291,292], with an annual growth rate of 5.7% for the period 2017–2022 [293]. Prodigiosin and violacein (chemical standards) are fetching about $5000 × 10^5^ per kg in the market [271]. Natural carotenoids (24%) are gaining a high market value of $350 to 7500 kg^−1^ than synthetic carotenoids (76%) with a value of $250–2000 kg^−1^ [294]. Astaxanthin and β-carotene are the highly demanded pigments globally with an expected market value of $225 and $309 million dollars by 2018, respectively [295]. Lutein is a xanthophyll pigment expected to gain a $308 million market value by 2018 [296]. The global market value of carotenoids is projected to reach up to 2.0 billion by 2026 [297]. According to the global phycobiliproteins market research report, market demand for phycobiliproteins is expected to rise by 2026. Currently, the phycobiliproteins (10 mg) price in Merck ranges from $200 to $270. A recent report has estimated the expected global dyes and pigments market value of $33.2 to 49.1 billion dollars by 2027 [298]. Indeed, 80 to 90% of the carotenoids supply in the market is fulfilled via chemical synthesis [299]. However, due to synthetic colorants’ side effects and the expensive pigment source of plants, microbial pigments have been gaining high demand in recent times. Therefore, finding potential promising microbes became a research interest in food and drug industries. For instance, yeast carotenoids’ market value has declined due to low dry weight production (0.40%) compared to algae, *Haematococcus* sp. (3.0%) [295]. In the current global population rise scenario, demand for edible microbial pigments as food colorants is expected to rise to fulfill the food industry requirements [300].

Microbial species with high biomass and pigment yield, including the genetically modified microbes, are highly interested in the current research. On the other hand, in view of the side effects posed with synthetic colorants [31,36,301], the scientific community has to reach the public through various social programs to make awareness about the importance of natural pigments and negative impacts of synthetic colorants on health. These awareness programs would save many lives from various health disorders, including life-threatening cancer.

## 13. Future Perspective

Microbial pigments demonstrated a wide variety of applications in food, drug, and textiles. These natural pigments can replace synthetic colorants and fulfill the emerging need on food colorants in the global market. Microbial pigments play an indirect role in the conservation of plants and animal resources by substituting them from pigment resources. Many researchers are restricted to pigments like prodigiosin, violacein, monascin, astaxanthin, lutein, and phycobiliproteins. Therefore, exploring other microbial pigments from different environments would offer novel and potential known pigment molecules for multifaceted applications. Research on microbial pigments would ultimately reveal the evolutionary lineages of origin of life and the dispersal of various chromophore-based phenomena in all lineages. Isolation and chemical characterization of microbial pigments are easier than non-pigmented microbes, whose compounds’ characterization is arduous and time-consuming. Thus, focusing on microbial pigments would garner more attention to research and development and their economic demand in various industries.

## Data Availability

Data sharing not applicable.

## References

[1] Scholes G.D., Mirkovic T., Turner D.B., Fassioli F., Buchleitner A. (2012). Solar light harvesting by energy transfer: From ecology to coherence. Energy Environ. Sci..

[2] Croce R., van Amerongen H. (2014). Natural strategies for photosynthetic light harvesting. Nat. Chem. Biol..

[3] Béja O., Spudich E.N., Spudich J.L., Leclerc M., DeLong E.F. (2001). Proteorhodopsin phototrophy in the ocean. Nature.

[4] Frigaard N.U., Martinez A., Mincer T.J., DeLong E.F. (2006). Proteorhodopsin lateral gene transfer between marine planktonic Bacteria and Archaea. Nature.

[5] Kamio M., Grimes T.V., Hutchins M.H., van Dam R., Derby C.D. (2010). The purple pigment aplysioviolin in sea hare ink deters predatory blue crabs through their chemical senses. Anim. Behav..

[6] Derby C.D. (2007). Escape by Inking and Secreting: Marine Molluscs Avoid Predators Through a Rich Array of Chemicals and Mechanisms. Biol. Bull..

[7] Derby C.D. (2014). Cephalopod Ink: Production, Chemistry, Functions and Applications. Mar. Drugs.

[8] Santos J.C., Coloma L.A., Cannatella D.C. (2003). Multiple, recurring origins of aposematism and diet specialization in poison frogs. Proc. Natl. Acad. Sci. USA.

[9] Ramesh C., Vinithkumar N.V., Kirubagaran R., Venil C.K., Dufossé L. (2019). Multifaceted applications of microbial pigments: Current knowledge, challenges and future directions for public health implications. Microorganisms.

[10] Davy S.H. (1799). Essays on Heat, Light, and the Combinations of Light, with a New Theory of Respiration. On the Generation of Oxygen Gas, and the Causes of the Colors of Organic Beings.

[11] Friedrich A.C. (1820). A Treatise on Adulterations of Food, and Culinary Poisons: Exhibiting the Fraudulent Sophistications of Bread, Beer, Wine, Spiritous Liquors, Tea, Coffee, Cream, Confectionery, Vinegar, Mustard, Pepper, Cheese, Olive Oil, Pickles, and Other Articles Employed in Domestic Economy and Methods of Detecting Them.

[12] Vandewalle G., Schmidt C., Albouy G., Sterpenich V., Darsaud A., Rauchs G., Berken P.-Y., Balteau E., Degueldre C., Luxen A. (2007). Brain Responses to Violet, Blue, and Green Monochromatic Light Exposures in Humans: Prominent Role of Blue Light and the Brainstem. PLoS ONE.

[13] Kurt S., Osueke K.K. (2014). The Effects of Color on the Moods of College Students. SAGE Open.

[14] Spence C. (2015). On the psychological impact of food colour. Flavour.

[15] Piqueras-Fiszman B., Giboreau A., Spence C. (2013). Assessing the influence of the color of the plate on the perception of a complex food in a restaurant setting. Flavour.

[16] Spence C., Levitan C.A., Shankar M.U., Zampini M. (2010). Does food color influence taste and flavor perception in humans?. Chemosens. Percept..

[17] Crameri F., Shephard G.E., Heron P.J. (2020). The misuse of colour in science communication. Nat. Commun..

[18] Olson J.A. (1989). Biological actions of carotenoids. J. Nutr..

[19] Downham A., Collins P. (2000). Colouring our foods in the last and next millennium. Int. J. Food Sci. Technol..

[20] Stoddard M.C., Eyster H.N., Hogan B.G., Morris D.H., Soucy E.R., Inouye D.W. (2020). Wild hummingbirds discriminate nonspectral colors. Proc. Natl. Acad. Sci. USA.

[21] Caves E.M., Green P.A., Zipple M.N., Bharath D., Peters S., Johnsen S., Nowicki S. (2020). Comparison of categorical color perception in two Estrildid finches. Am. Nat..

[22] Newsome A.G., Murphy B.T., Van Breemen R. (2013). Isolation and characterization of natural blue pigments from underexplored sources. ACS Symp. Ser..

[23] Venil C.K., Dufossé L., Devi P.R. (2020). Bacterial Pigments: Sustainable Compounds With Market Potential for Pharma and Food Industry. Front. Sustain. Food Syst..

[24] Dufossé L., Molina G., Gupta V.K., Singh B.N., Gathergood N. (2020). Research, Development, and Production of Microalgal and Microbial Biocolorants. Bioprocessing for Biomolecules Production.

[25] Ramesh C., Vinithkumar N.V., Kirubagaran R. (2019). Marine pigmented bacteria: A prospective source of antibacterial compounds. J. Nat. Sci. Biol. Med..

[26] Nawaz A., Chaudhary R., Shah Z., Dufossé L., Fouillaud M., Mukhtar H., ul Haq I. (2021). An overview on industrial and medical applications of bio-pigments synthesized by marine bacteria. Microorganisms.

[27] Rajapaksha G.K.M., Wansapala M.A.J., Silva A.B.G. (2017). Detection of Synthetic Colours in Selected Foods & Beverages Available in Colombo District, Sri Lanka. Int. J. Sci. Res..

[28] Saleem N., Umar Z.N., Khan S.I. (2013). Survey on the use of synthetic Food Colors in Food Samples procured from different educational institutes of Karachi city. J. Trop. Life. Sci..

[29] Okafor S.N., Obonga W., Ezeokonkwo M.A., Nurudeen J., Orovwigho U., Ahiabuike J. (2016). Assessment of the Health implications of Synthetic and Natural Food Colourants-A Critical Review. UK J. Pharm. Biosci..

[30] Babitha S., Nigam P.S., Pandey A. (2009). Microbial pigments. Biotechnology for Agro-Industrial Residues Utilisation: Utilisation of Agro-Residues.

[31] Burrows A.J.D. (2009). Palette of our palates: A brief history of food coloring and its regulation. Compr. Rev. Food Sci. Food Saf..

[32] Lellis B., Fávaro-Polonio C.Z., Pamphile J.A., Polonio J.C. (2019). Effects of textile dyes on health and the environment and bioremediation potential of living organisms. Biotechnol. Res. Innov..

[33] Saini R.D. (2017). Textile organic dyes: Polluting effects and elimination methods from textile waste water. Int. J. Chem. Eng. Res..

[34] Berradi M., Hsissou R., Khudhair M., Assouag M., Cherkaoui O., El Bachiri A., El Harfi A. (2019). Textile finishing dyes and their impact on aquatic environs. Heliyon.

[35] Jamee R., Siddique R. (2019). Biodegradation of synthetic dyes of textile effluent by microorganisms: An environmentally and economically sustainable approach. Eur. J. Microbiol. Immunol..

[36] Roy A.K.C. (2018). Eco-friendly dyes and dyeing. Adv. Mat. Technol. Environ..

[37] Puvaneswari N., Muthukrishnan J., Gunasekaran P. (2006). Toxicity assessment and microbial degradation of azo dyes. Indian J. Exp. Biol..

[38] Mumtaz R., Bashir S., Numan M., Shinwari Z.K., Ali M. (2019). Pigments from soil bacteria and their therapeutic properties: A mini review. Curr. Microbiol..

[39] Pailliè-Jiménez M.E., Stincone P., Brandelli A. (2020). Natural pigments of microbial origin. Front. Sustain. Food Syst..

[40] Venil C.K., Velmurugan P., Dufossé L., Devi P.R., Ravi A.V. (2020). Fungal pigments: Potential coloring compounds for wide ranging applications in textile dyeing. J. Fungi.

[41] Sen T., Barrow C.J., Deshmukh S.K. (2019). Microbial pigments in the food industry—Challenges and the way forward. Front. Nutr..

[42] Narsing Rao M.P., Xiao M., Li W.J. (2017). Fungal and bacterial pigments: Secondary metabolites with wide applications. Front. Microbiol..

[43] Darshan N., Manonmani H.K. (2015). Prodigiosin and its potential applications. J. Food Sci. Technol..

[44] De Carvalho J.C., Bicas J.L., Fernández D.E.R., Woiciechowski A.L., Medeiros A.B.P., Soccol C.R., Bicas J.L., Maróstica M.R., Pastore G.M. (2016). Natural colorants from microorganisms. Biotechnological Production of Natural Ingredients for Food Industry.

[45] Numan M., Bashir S., Mumtaz R., Tayyab S., Rehman N.U., Khan A.L., Shinwari Z.K., Al-Harrasi A. (2018). Therapeutic applications of bacterial pigments: A review of current status and future opportunities. 3 Biotech.

[46] Kenaley C.P. (2010). Comparative Innervation of Cephalic Photophores of the Loosejaw Dragonfishes (Teleostei:Stomiiformes:Stomiidae): Evidence for Parallel Evolution of. J. Morphol..

[47] Kenaley C.P., DeVaney S.C., Fjeran T.T. (2014). The complex evolutionary history of seeing red: Molecular phylogeny and the evolution of an adaptive visual system in deep-sea dragonfishes (Stomiiformes: Stomiidae). Evolution.

[48] Roy A., Pittman M., Saitta E.T., Kaye T.G., Xu X. (2020). Recent advances in amniote palaeocolour reconstruction and a framework for future research. Biol. Rev..

[49] Lindgren J. (2016). Fossil pigments. Curr. Biol..

[50] Colleary C., Dolocan A., Gardner J., Singh S., Wuttke M., Rabenstein R., Habersetzer J., Schaal S., Feseha M., Clemens M. (2015). Chemical, experimental, and morphological evidence for diagenetically altered melanin in exceptionally preserved fossils. Proc. Natl. Acad. Sci. USA.

[51] Blumer M. (1960). Pigments of a fossil echinoderm. Nature.

[52] Zhang F., Kearns S.L., Orr P.J., Benton M.J., Zhou Z., Johnson D., Xu X., Wang X. (2010). Fossilized melanosomes and the colour of Cretaceous dinosaurs and birds. Nature.

[53] Babarovic F., Puttick M.N., Zaher M., Learmonth E., Gallimore E.J., Smithwick F.M., Mayr G., Vinther J. (2019). Characterization of melanosomes involved in the production of non-iridescent structural feather colours and their detection in the fossil record. J. R. Soc. Interface.

[54] Pérez-Diez S., Fernández-Menéndez L.J., Morillas H., Martellone A., De Nigris B., Osanna M., Bordel N., Caruso F., Madariaga J.M., Maguregui M. (2020). Elucidation of the chemical role of the pyroclastic materials on the state of conservation of mural paintings from Pompeii. Angew. Chemie.

[55] Siddall R. (2018). Mineral pigments in archaeology: Their analysis and the range of available materials. Minerals.

[56] Reiche I. (2019). Mineral pigments: The colourful palette of nature. Eur. Mineral. Union Notes Mineral..

[57] Barnett J.R., Miller S., Pearce E. (2006). Colour and art: A brief history of pigments. Opt. Laser Technol..

[58] Zilhão J., Angelucci D.E., Badal-García E., d’Errico F., Daniel F., Dayet L., Douka K., Higham T.F.G., Martínez-Sánchez M.J., Montes-Bernárdez R. (2010). Symbolic use of marine shells and mineral pigments by Iberian Neandertals. Proc. Natl. Acad. Sci. USA.

[59] Aubert M., Lebe R., Oktaviana A.A., Tang M., Burhan B., Hamrullah, Jusdi A., Abdullah, Hakim B., Zhao J.X. (2019). Earliest hunting scene in prehistoric art. Nature.

[60] Kurniawan R., Kadja G.T.M., Setiawan P., Burhan B., Oktaviana A.A., Rustan, Hakim B., Aubert M., Brumm A., Ismunandar (2019). Chemistry of prehistoric rock art pigments from the Indonesian island of Sulawesi. Microchem. J..

[61] Martins N., Roriz C.L., Morales P., Barros L., Ferreira I.C.F.R. (2016). Food colorants: Challenges, opportunities and current desires of agro-industries to ensure consumer expectations and regulatory practices. Trends Food Sci. Technol..

[62] Smith J., Hong-Shum L. (2011). Food Additives Data Book.

[63] Sengar G., Kumar H. (2014). Food caramels: A review. J. Food. Sci. Technol..

[64] Velmurugan P., Venil C.K., Veera Ravi A., Dufossé L. (2020). Marine bacteria is the cell factory to produce bioactive pigments: A prospective pigment source in the ocean. Front. Sustain. Food Syst..

[65] Yutin N., Koonin E.V. (2012). Proteorhodopsin genes in giant viruses. Biol. Direct.

[66] Slamovits C.H., Okamoto N., Burri L., James E.R., Keeling P.J. (2011). A bacterial proteorhodopsin proton pump in marine eukaryotes. Nat. Commun..

[67] Cao W., McCallum N.C., Ni Q.Z., Li W., Boyce H., Mao H., Zhou X., Sun H., Thompson M.P., Battistella C. (2020). Selenomelanin: An abiotic selenium analogue of pheomelanin. J. Am. Chem. Soc..

[68] Kientz B., Luke S., Vukusic P., Péteri R., Beaudry C., Renault T., Simon D., Mignot T., Rosenfeld E. (2016). A unique self-organization of bacterial sub-communities creates iridescence in *Cellulophaga lytica* colony biofilms. Sci. Rep..

[69] Vinther J., Briggs D.E.G., Clarke J., Mayr G., Prum R.O. (2010). Structural coloration in a fossil feather. Biol. Lett..

[70] Buchan A., Neidle E.L., Moran M.A. (2004). Diverse organization of genes of the β-ketoadipate pathway in members of the marine *Roseobacter* lineage. Appl. Environ. Microbiol..

[71] Afra S., Makhdoumi A., Matin M.M., Feizy J. (2017). A novel red pigment from marine *Arthrobacter* sp. G20 with specific anticancer activity. J. Appl. Microbiol..

[72] Choi S.Y., Lim S., Cho G., Kwon J., Mun W., Im H., Mitchell R.J. (2020). *Chromobacterium violaceum* delivers violacein, a hydrophobic antibiotic, to other microbes in membrane vesicles. Environ. Microbiol..

[73] Borić M., Danevčič T., Stopar D. (2011). Prodigiosin from *Vibrio* sp. DSM 14379; a new UV-protective pigment. Microb. Ecol..

[74] Matz C., Deines P., Boenigk J., Arndt H., Eberl L., Kjelleberg S., Jürgens K. (2004). Impact of violacein-producing bacteria on survival and feeding of bacterivorous nanoflagellates. Appl. Environ. Microbiol..

[75] Brucker R.M., Harris R.N., Schwantes C.R., Gallaher T.N., Flaherty D.C., Lam B.A., Minbiole K.P.C. (2008). Amphibian chemical defense: Antifungal metabolites of the microsymbiont *Janthinobacterium lividum* on the salamander *Plethodon cinereus*. J. Chem. Ecol..

[76] Harris R.N., Brucker R.M., Walke J.B., Becker M.H., Schwantes C.R., Flaherty D.C., Lam B.A., Woodhams D.C., Briggs C.J., Vredenburg V.T. (2009). Skin microbes on frogs prevent morbidity and mortality caused by a lethal skin fungus. ISME J..

[77] Becker M.H., Brucker R.M., Schwantes C.R., Harris R.N., Minbiole K.P.C. (2009). The bacterially produced metabolite violacein is associated with survival of amphibians infected with a lethal fungus. Appl. Environ. Microbiol..

[78] Pierson L.S., Pierson E.A. (2010). Metabolism and function of phenazines in bacteria: Impacts on the behavior of bacteria in the environment and biotechnological processes. Appl. Microbiol. Biotechnol..

[79] Visca P., Imperi F., Lamont I.L. (2007). Pyoverdine siderophores: From biogenesis to biosignificance. Trends Microbiol..

[80] Franks A., Haywood P., Holmström C., Egan S., Kjelleberg S., Kumar N. (2005). Isolation and structure elucidation of a novel yellow pigment from the marine bacterium *Pseudoalteromonas tunicata*. Molecules.

[81] Egan S., James S., Holmström C., Kjelleberg S. (2002). Correlation between pigmentation and antifouling compounds produced by *Pseudoalteromonas tunicata*. Environ. Microbiol..

[82] Cude W.N., Mooney J., Tavanaei A.A., Hadden M.K., Frank A.M., Gulvik C.A., May A.L., Buchan A. (2012). Production of the antimicrobial secondary metabolite indigoidine contributes to competitive surface colonization by the marine roseobacter *Phaeobacter* sp. strain Y4I. Appl. Environ. Microbiol..

[83] Núñez-Pons L., Avila C., Romano G., Verde C., Giordano D. (2018). UV-protective compounds in marine organisms from the southern ocean. Mar. Drugs.

[84] Plonka P.M., Grabacka M. (2006). Melanin synthesis in microorganisms—Biotechnological and medical aspects. Acta Biochim. Pol..

[85] Kotob S.I., Coon S.L., Quintero E.J., Weiner R.M. (1995). Homogentisic acid is the primary precursor of melanin synthesis in *Vibrio cholerae*, a *Hyphomonas* strain, and *Shewanella colwelliana*. Appl. Environ. Microbiol..

[86] Ivanova E.P., Kiprianova E.A., Mikhailov V.V., Levanova G.F., Garagulya A.D., Gorshkova N.M., Yumoto N., Yoshikawa S. (1996). Characterization and identification of marine *Alteromonas nigrifaciens* strains and emendation of the description. Int. J. Syst. Bacteriol..

[87] Kahng H.Y., Chung B.S., Lee D.H., Jung J.S., Park J.H., Jeon C.O. (2009). *Cellulophaga tyrosinoxydans* sp. nov., a tyrosinase-producing bacterium isolated from seawater. Int. J. Syst. Evol. Microbiol..

[88] Coyne V.E., Al-Harthi L. (1992). Induction of melanin biosynthesis in *Vibrio cholerae*. Appl. Environ. Microbiol..

[89] Gessler N.N., Egorova A.S., Belozerskaya T.A. (2013). Fungal anthraquinones. Appl. Biochem. Microbiol..

[90] Cordero R.J.B., Casadevall A. (2017). Functions of fungal melanin beyond virulence. Fungal Biol. Rev..

[91] Mathews M.M., Sistrom W.R. (1959). Function of carotenoid pigments in non-photosynthetic bacteria. Nature.

[92] Sakimoto K.K., Wong A.B., Yang P. (2016). Self-photosensitization of nonphotosynthetic bacteria for solar-to-chemical production. Science.

[93] Krinsky N.I. (1978). Non-photosynthetic functions of carotenoids. Philos. Trans. R. Soc. London B.

[94] Agogué H., Joux F., Obernosterer I., Lebaron P. (2005). Resistance of marine bacterioneuston to solar radiation. Appl. Environ. Microbiol..

[95] Hermansson M., Jones G.W., Kjelleberg S. (1987). Frequency of antibiotic and heavy metal resistance, pigmentation, and plasmids in bacteria of the marine air-water interface. Appl. Environ. Microbiol..

[96] Mandelli F., Miranda V.S., Rodrigues E., Mercadante A.Z. (2012). Identification of carotenoids with high antioxidant capacity produced by extremophile microorganisms. World J. Microbiol. Biotechnol..

[97] Dieser M., Greenwood M., Foreman C.M. (2010). Carotenoid pigmentation in Antarctic heterotrophic bacteria as a strategy to withstand environmental stresses. Arctic, Antarct. Alp. Res..

[98] Marizcurrena J.J., Cerdá M.F., Alem D., Castro-Sowinski S. (2019). Living with Pigments: The Colour Palette of Antarctic Life. The Ecological Role of Micro-Organisms in the Antarctic Environment.

[99] Albuquerque L., Da Costa M.S., Rosenberg E., DeLong E.F., Lory S., Stackebrandt E., Thompson F. (2014). The family thermaceae. The Prokaryotes: Other Major Lineages of Bacteria and The Archaea.

[100] Rodrigo-Baños M., Garbayo I., Vílchez C., Bonete M.J., Martínez-Espinosa R.M. (2015). Carotenoids from Haloarchaea and their potential in biotechnology. Mar. Drugs.

[101] Moliné M., Flores M.R., Libkind D., Del Carmen Diéguez M., Farías M.E., Van Broock M. (2010). Photoprotection by carotenoid pigments in the yeast *Rhodotorula mucilaginosa*: The role of torularhodin. Photochem. Photobiol. Sci..

[102] Moliné M., Libkind D., del Carmen Diéguez M., van Broock M. (2009). Photoprotective role of carotenoids in yeasts: Response to UV-B of pigmented and naturally-occurring albino strains. J. Photochem. Photobiol. B Biol..

[103] Rastogi R.P., Sonani R.R., Madamwar D. (2017). UV photoprotectants from algae-synthesis and bio-functionalities. Algal Green Chemistry: Recent Progress in Biotechnology.

[104] Gastineau R., Hardivillier Y., Leignel V., Tekaya N., Morançais M., Fleurence J., Davidovich N., Jacquette B., Gaudin P., Hellio C. (2012). Greening effect on oysters and biological activities of the blue pigments produced by the diatom *Haslea karadagensis* (Naviculaceae). Aquaculture.

[105] Falaise C., James A., Travers M.A., Zanella M., Badawi M., Mouget J.L. (2019). Complex relationships between the blue pigment marennine and marine bacteria of the genus *Vibrio*. Mar. Drugs.

[106] Turcotte F., Mouget J.L., Genard B., Lemarchand K., Deschênes J.S., Tremblay R. (2016). Prophylactic effect of *Haslea ostrearia* culture supernatant containing the pigment marennine to stabilize bivalve hatchery production. Aquat. Living Resour..

[107] Jitmuang A. (2008). Human *Chromobacterium violaceum* infection in Southeast Asia: Case reports and literature review. Southeast Asian J. Trop. Med. Public Health.

[108] Oh W.T., Giri S.S., Yun S., Kim H.J., Kim S.G., Kim S.W., Kang J.W., Han S.J., Kwon J., Jun J.W. (2019). *Janthinobacterium lividum* as an emerging pathogenic bacterium affecting rainbow trout (*Oncorhynchus mykiss*) fisheries in Korea. Pathogens.

[109] Petersen L.M., Tisa L.S. (2013). Friend or foe? a review of the mechanisms that drive *Serratia* towards diverse lifestyles. Can. J. Microbiol..

[110] Grimont F., Grimont P.A.D., Dworkin M., Falkow S., Rosenberg E., Schleifer K.H., Stackebrandt E. (2006). The genus *Serratia*. The Prokaryotes.

[111] Mahlen S.D. (2011). *Serratia* infections: From military experiments to current practice. Clin. Microbiol. Rev..

[112] Sharmin S., Kamal S.M. (2019). Review on *Chromobacterium violaceum*, a rare but fatal bacteria needs special clinical attention. Anwer Khan Mod. Med. Coll. J..

[113] Zhou W., Li J.H., Chen J., Liu X.Y., Xiang T.T., Zhang L., Wan Y.J. (2016). The red pigment prodigiosin is not an essential virulence factor in entomopathogenic *Serratia marcescens*. J. Invertebr. Pathol..

[114] Nosanchuk J.D., Casadevall A. (2003). The contribution of melanin to microbial pathogenesis. Cell. Microbiol..

[115] Kang D., Revtovich A.V., Chen Q., Shah K.N., Cannon C.L., Kirienko N.V. (2019). Pyoverdine-dependent virulence of *Pseudomonas aeruginosa* isolates from cystic fibrosis patients. Front. Microbiol..

[116] Wang Y.Z., Ju X.L., Zhou Y.G. (2005). The variability of citrinin production in Monascus type cultures. Food Microbiol..

[117] Mapari S.A.S., Meyer A.S., Thrane U., Frisvad J.C. (2009). Identification of potentially safe promising fungal cell factories for the production of polyketide natural food colorants using chemotaxonomic rationale. Microb. Cell Fact..

[118] Hakvåg S., Fjærvik E., Klinkenberg G., Borgos S.E.F., Josefsen K.D., Ellingsen T.E., Zotchev S.B. (2009). Violacein-producing Collimonas sp. from the sea surface microlayer of costal waters in Trøndelag, Norway. Mar. Drugs.

[119] Urbanczyk H., Ast J.C., Kaeding A.J., Oliver J.D., Dunlap P.V. (2008). Phylogenetic analysis of the incidence of lux gene horizontal transfer in Vibrionaceae. J. Bacteriol..

[120] Ziebuhr W., Ohlsen K., Karch H., Korhonen T., Hacker J. (1999). Evolution of bacterial pathogenesis. Cell. Mol. Life Sci..

[121] Choi S.Y., Yoon K.H., Lee J.I., Mitchell R.J. (2015). Violacein: Properties and production of a versatile bacterial pigment. Biomed Res. Int..

[122] Kobayashi C., Karakas A.I., Lugaro M. (2020). The Origin of Elements from Carbon to Uranium. Astrophys. J..

[123] Wang S., Xu F., Zhan J., Singh O.V. (2017). Introduction of Natural Pigments from Microorganisms. Bio-pigmentation and Biotechnological Implementations.

[124] Bandaranayake W.M. (2006). The nature and role of pigments of marine invertebrates. Nat. Prod. Rep..

[125] Leavitt P.R., Hodgson D.A., Smol J.P., Birks H.J.B., Last W.M. (2001). Sedimentary Pigments. Tracking Environmental Change Using Lake Sediments. Volume 3: Terrestrial, Algal, and Siliceous Indicators.

[126] Sanger J.E. (1988). Fossil pigments in paleoecology and paleolimnology. Palaeogeogr. Palaeoclimatol. Palaeoecol..

[127] Reuss N. (2005). Sediment Pigments as Biomarkers of Environmental Change. Ph.D. Thesis.

[128] Hashizume H., Valaškova M., Martynkova G.S. (2012). Role of Clay Minerals in Chemical Evolution and the Origins of Life. Clay Minerals in Nature—Their Characterization, Modification and Application.

[129] Gómez F., Cavalazzi B., Rodríguez N., Amils R., Ori G.G., Olsson-Francis K., Escudero C., Martínez J.M., Miruts H. (2019). Ultra-small microorganisms in the polyextreme conditions of the Dallol volcano, Northern Afar, Ethiopia. Sci. Rep..

[130] Jordan S.F., Rammu H., Zheludev I.N., Hartley A.M., Maréchal A., Lane N. (2019). Promotion of protocell self-assembly from mixed amphiphiles at the origin of life. Nat. Ecol. Evol..

[131] Kim S.C., Zhou L., Zhang W., O’Flaherty D.K., Rondo-Brovetto V., Szostak J.W. (2020). A model for the emergence of RNA from a prebiotically plausible mixture of ribonucleotides, arabinonucleotides, and 2′-deoxynucleotides. J. Am. Chem. Soc..

[132] Belilla J., Moreira D., Jardillier L., Reboul G., Benzerara K., López-García J.M., Bertolino P., López-Archilla A.I., López-García P. (2019). Hyperdiverse archaea near life limits at the polyextreme geothermal Dallol area. Nat. Ecol. Evol..

[133] Dautel D.R., Champion J.A. (2020). Protein vesicles self-assembled from functional globular proteins with different charge and size. Biomacromolecules.

[134] Hourdez S., Weber R.E. (2005). Molecular and functional adaptations in deep-sea hemoglobins. J. Inorg. Biochem..

[135] Terakita A. (2005). The opsins. Genome Biol..

[136] Yizhar O., Fenno L., Zhang F., Hegemann P., Deisseroth K. (2011). Microbial Opsins A Family of Single-Component Tools for Optical Control of Neural Activity. Cold Spring Harb. Protoc..

[137] Davies W.I.L., Collin S.P., Hunt D.M. (2012). Molecular ecology and adaptation of visual photopigments in craniates. Mol. Ecol..

[138] Spudich J.L., Yang C.S., Jung K.H., Spudich E.N. (2000). Retinylidene proteins: Structures and functions from archaea to humans. Annu. Rev. Cell Dev. Biol..

[139] Kannaujiya V.K., Shanthy S., Sinha R.P., Kannaujiya V.K., Shanthy S., Sinha R.P. (2017). Evolution of phycobiliproteins. Phycobiliproteins: Recent developments and future applications.

[140] Green B.R. (2007). Evolution of Light-Harvesting Antennas in an Oxygen World. Evolution of Primary Producers in the Sea.

[141] Apt K.E., Collier J.L., Grossman A.R. (1995). Evolution of the phycobiliproteins. J. Mol. Biol..

[142] Greenwold M.J., Cunningham B.R., Lachenmyer E.M., Pullman J.M., Richardson T.L., Dudycha J.L. (2019). Diversification of light capture ability was accompanied by the evolution of phycobiliproteins in cryptophyte algae. Proc. R. Soc. B Biol. Sci..

[143] Palenik B., Haselkorn R. (1992). Multiple evolutionary origins of prochlorophytes, the chlorophyll b-containing prokaryotes. Nature.

[144] Cardona T. (2016). Origin of bacteriochlorophyll *a* and the early diversification of photosynthesis. PLoS ONE.

[145] Xu J., Chmela V., Green N.J.J., Russell D.A.A., Janicki M.J.J., Góra R.W.W., Szabla R., Bond A.D.D., Sutherland J.D.D. (2020). Selective prebiotic formation of RNA pyrimidine and DNA purine nucleosides. Nature.

[146] Horning D.P., Joyce G.F. (2016). Amplification of RNA by an RNA polymerase ribozyme. Proc. Natl. Acad. Sci. USA.

[147] Yi R., Tran Q.P., Ali S., Yoda I., Adam Z.R., Cleaves H.J., Fahrenbach A.C. (2020). A continuous reaction network that produces RNA precursors. Proc. Natl. Acad. Sci. USA.

[148] Bernhardt H.S. (2012). The RNA world hypothesis: The worst theory of the early evolution of life (except for all the others) a. Biol. Direct.

[149] Teichert J.S., Kruse F.M., Trapp O. (2019). Direct prebiotic pathway to DNA nucleosides. Angew. Chem. Int. Ed..

[150] Wołos A., Roszak R., Żądło-Dobrowolska A., Beker W., Mikulak-Klucznik B., Spólnik G., Dygas M., Szymkuć S., Grzybowski B.A. (2020). Synthetic connectivity, emergence, and self-regeneration in the network of prebiotic chemistry. Science.

[151] Mehr S.H.M., Craven M., Leonov A.I., Keenan G., Cronin L. (2020). A universal system for digitization and automatic execution of the chemical synthesis literature. Science.

[152] Harris A.K.P., Williamson N.R., Slater H., Cox A., Abbasi S., Foulds I., Simonsen H.T., Leeper F.J., Salmond G.P.C. (2004). The *Serratia* gene cluster encoding biosynthesis of the red antibiotic, prodigiosin, shows species- and strain-dependent genome context variation. Microbiology.

[153] Williamson N.R., Fineran P.C., Leeper F.J., Salmond G.P.C. (2006). The biosynthesis and regulation of bacterial prodiginines. Nat. Rev. Microbiol..

[154] Cerdeño A.M., Bibb M.J., Challis G.L. (2001). Analysis of the prodiginine biosynthesis gene cluster of *Streptomyces coelicolor* A3(2): New mechanisms for chain initiation and termination in modular multienzymes. Chem. Biol..

[155] Kim D., Park Y.K., Lee J.S., Kim J.F., Jeong H., Kim B.S., Lee C.H. (2006). Analysis of a prodigiosin biosynthetic gene cluster from the marine bacterium *Hahella chejuensis* KCTC 2396. J. Microbiol. Biotechnol..

[156] Sánchez C., Braña A.F., Méndez C., Salas J.A. (2006). Reevaluation of the violacein biosynthetic pathway and its relationship to indolocarbazole biosynthesis. ChemBioChem.

[157] Zhang J.J., Tang X., Zhang M., Nguyen D., Moore B.S. (2017). Broad-host-range expression reveals native and host regulatory elements that influence heterologous antibiotic production in Gram-negative bacteria. MBio.

[158] Burke C., Thomas T., Egan S., Kjelleberg S. (2007). The use of functional genomics for the identification of a gene cluster encoding for the biosynthesis of an antifungal tambjamine in the marine bacterium *Pseudoalteromonas tunicata*: Brief report. Environ. Microbiol..

[159] Hunter R.C., Newman D.K. (2010). A putative ABC transporter, hatABCDE, is among molecular determinants of pyomelanin production in *Pseudomonas aeruginosa*. J. Bacteriol..

[160] Wiemann P., Willmann A., Straeten M., Kleigrewe K., Beyer M., Humpf H.U., Tudzynski B. (2009). Biosynthesis of the red pigment bikaverin in *Fusarium fujikuroi*: Genes, their function and regulation. Mol. Microbiol..

[161] Chen W., He Y., Zhou Y., Shao Y., Feng Y., Li M., Chen F. (2015). Edible filamentous fungi from the species *Monascus*: Early taditional fermentations, modern molecular biology, and future genomics. Compr. Rev. Food Sci. Food Saf..

[162] Chen W., Chen R., Liu Q., He Y., He K., Ding X., Kang L., Guo X., Xie N., Zhou Y. (2017). Orange, red, yellow: Biosynthesis of azaphilone pigments in *Monascus* fungi. Chem. Sci..

[163] Balakrishnan B., Karki S., Chiu S.H., Kim H.J., Suh J.W., Nam B., Yoon Y.M., Chen C.C., Kwon H.J. (2013). Genetic localization and in vivo characterization of a *Monascus* azaphilone pigment biosynthetic gene cluster. Appl. Microbiol. Biotechnol..

[164] Kwon H.J., Balakrishnan B., Kim Y.K. (2016). Some *Monascus purpureus* genomes lack the monacolin K biosynthesis locus. J. Appl. Biol. Chem..

[165] Nishida Y., Adachi K., Kasai H., Shizuri Y., Shindo K., Sawabe A., Komemushi S., Miki W., Misawa N. (2005). Elucidation of a carotenoid biosynthesis gene cluster encoding a novel enzyme, 2,2′-β-hydroxylase, from *Brevundimonas* sp. strain SD212 and combinatorial biosynthesis of new or rare xanthophylls. Appl. Environ. Microbiol..

[166] Gao Z., Li Y., Wu G., Li G., Sun H., Deng S., Shen Y., Chen G., Zhang R., Meng C. (2015). Transcriptome analysis in *Haematococcus pluvialis*: Astaxanthin induction by salicylic acid (SA) and jasmonic acid (JA). PLoS ONE.

[167] Xu X., Tian L., Xu J., Xie C., Jiang L., Huang H. (2018). Analysis and expression of the carotenoid biosynthesis genes from *Deinococcus wulumuqiensis* R12 in engineered Escherichia coli. AMB Express.

[168] Álvarez V., Rodríguez-Sáiz M., de la Fuente J.L., Gudiña E.J., Godio R.P., Martín J.F., Barredo J.L. (2006). The crtS gene of *Xanthophyllomyces dendrorhous* encodes a novel cytochrome-P450 hydroxylase involved in the conversion of β-carotene into astaxanthin and other xanthophylls. Fungal Genet. Biol..

[169] Styczynski M., Rogowska A., Gieczewska K., Garstka M., Szakiel A., Dziewit L. (2020). Genome-based insights into the production of carotenoids by Antarctic bacteria, *Planococcus* sp. ANT_H30 and *Rhodococcus* sp. ANT_H53B. Molecules.

[170] Lee P.C., Schmidt-Dannert C. (2002). Metabolic engineering towards biotechnological production of carotenoids in microorganisms. Appl. Microbiol. Biotechnol..

[171] Elleuch F., Hlima H.B., Barkallah M., Baril P., Abdelkafi S., Pichon C., Fendri I. (2019). Carotenoids overproduction in *Dunaliella* sp.: Transcriptional changes and new insights through lycopene cyclase regulation. Appl. Sci..

[172] Landolfo S., Ianiri G., Camiolo S., Porceddu A., Mulas G., Chessa R., Zara G., Mannazzu I. (2018). CAR gene cluster and transcript levels of carotenogenic genes in *Rhodotorula mucilaginosa*. Microbiology (UK).

[173] Giri A.V., Anandkumar N., Muthukumaran G., Pennathur G. (2004). A novel medium for the enhanced cell growth and production of prodigiosin from *Serratia marcescens* isolated from soil. BMC Microbiol..

[174] Wei Y.-H., Chen W.-C. (2005). Enhanced production of prodigiosin-like pigment from *Serratia marcescens* SMΔR by medium improvement and oil-supplementation strategies. J. Biosci. Bioeng..

[175] Wang S.L., Wang C.Y., Yen Y.H., Liang T.W., Chen S.Y., Chen C.H. (2012). Enhanced production of insecticidal prodigiosin from *Serratia marcescens* TKU011 in media containing squid pen. Process Biochem..

[176] De Araújo H.W.C., Fukushima K., Takaki G.M.C. (2010). Prodigiosin production by *Serratia marcescens* UCP 1549 using renewable-resources as a low cost substrate. Molecules.

[177] Aruldass C.A., Venil C.K., Zakaria Z.A., Ahmad W.A. (2014). Brown sugar as a low-cost medium for the production of prodigiosin by locally isolated Serratia marcescens UTM1. Int. Biodeterior. Biodegrad..

[178] Sumathi C., Mohanapriya D., Swarnalatha S., Dinesh M.G., Sekaran G. (2014). Production of prodigiosin using tannery fleshing and evaluating its pharmacological effects. Sci. World J..

[179] Kurbanoglu E.B., Ozdal M., Ozdal O.G., Algur O.F. (2015). Enhanced production of prodigiosin by Serratia marcescens MO-1 using ram horn peptone. Braz. J. Microbiol..

[180] Xia S., Veony E., Yang Q. (2018). Kitchen waste as a novel available substrate for prodigiosin production by *Serratia marcescense*. IOP Conf. Ser. Earth Environ. Sci..

[181] Arivizhivendhan K.V., Mahesh M., Boopathy R., Swarnalatha S., Regina Mary R., Sekaran G. (2018). Antioxidant and antimicrobial activity of bioactive prodigiosin produces from *Serratia marcescens* using agricultural waste as a substrate. J. Food Sci. Technol..

[182] Suryawanshi R.K., Patil C.D., Borase H.P., Salunke B.K., Patil S.V. (2014). Studies on production and biological potential of prodigiosin by *Serratia marcescens*. Appl. Biochem. Biotechnol..

[183] Xia Y., Wang G., Lin X., Song X., Ai L. (2016). Solid-state fermentation with *Serratia marcescens* Xd-1 enhanced production of prodigiosin by using bagasse as an inertia matrix. Ann. Microbiol..

[184] Aniyan N.B., Thomas S.K. (2019). Solid state fermentation for prodigiosin production using *Serratia marcescens*. Int. Res. J. Eng. Technol..

[185] Ahmad W.A., Ahmad W.Y.W., Zakaria Z.A., Yusof N.Z. (2012). Optimization of pigment production: Case of *Chromobacterium violaceum* and *Serratia marcescens*. Application of Bacterial Pigments as Colorant.

[186] Ahmad W.A., Venil C.K., Aruldass C.A., Liong M.T. (2015). Production of violacein by *Chromobacterium violaceum* grown in liquid pineapple waste: Current scenario. Beneficial Microorganisms in Agriculture, Aquaculture and Other Areas, Microbiology Monographs.

[187] El-Fouly M.Z., Sharaf A.M., Shahin A.A.M., El-Bialy H.A., Omara A.M.A. (2015). Biosynthesis of pyocyanin pigment by *Pseudomonas aeruginosa*. J. Radiat. Res. Appl. Sci..

[188] Timotius K.H. (2005). The influence of tapioca on the growth, the activity of glucoamylase and pigment production of *Monascus purpureus* UKSW 40 in soybean-soaking wastewater. World J. Microbiol. Biotechnol..

[189] Babitha S., Soccol C.R., Pandey A. (2007). Solid-state fermentation for the production of *Monascus* pigments from jackfruit seed. Bioresour. Technol..

[190] Babitha S., Soccol C.R., Pandey A. (2006). Jackfruit seed—A novel substrate for the production of *Monascus* pigments through solid-state fermentation. Food Technol. Biotechnol..

[191] Silveira S.T., Daroit D.J., Brandelli A. (2008). Pigment production by *Monascus purpureus* in grape waste using factorial design. Food Sci. Technol..

[192] Nimnoi P., Lumyong S. (2011). Improving solid-state fermentation of *Monascus purpureus* on agricultural products for pigment production. Food Bioprocess Technol..

[193] Velmurugan P., Hur H., Balachandar V., Kamala-Kannan S., Lee K.J., Lee S.M., Chae J.C., Shea P.J., Oh B.T. (2011). *Monascus* pigment production by solid-state fermentation with corn cob substrate. J. Biosci. Bioeng..

[194] Subhasree R.S., Dinesh Babu P., Vidyalakshmi R., Mohan V.C. (2011). Effect of carbon and nitrogen sources on stimulation of pigment production by Monascus purpureuson jackfruit seeds. Int. J. Microbiol. Res..

[195] Vidyalakshmi R., Paranthaman R., Murugesh S., Singaravadivel K. (2009). Microbial bioconversion of rice broken to food grade pigments. Glob. J. Biotechnol. Biochem..

[196] Dikshit R., Tallapragada P. (2011). *Monascus purpureus*: A potential source for natural pigment production. J. Microbiol. Biotechnol..

[197] Said F.M., Chisti Y., Brooks J. (2010). The effects of forced aeration and initial moisture level on red pigment and biomass production by *Monascus ruber* in packed bed solid state fermentation. Int. J. Environ. Sci. Dev..

[198] Srianta I., Novita Y., Kusumawati N. (2012). Production of monascus pigments on durian seed: Effect of supplementation of carbon source. J. Pure Appl. Microbiol..

[199] Silveira S.T., Daroit D.J., Sant’Anna V., Brandelli A. (2013). Stability modeling of red pigments produced by *Monascus purpureus* in submerged cultivations with sugarcane bagasse. Food Bioprocess Technol..

[200] Srivastav P., Yadav V.K., Govindasamy S., Chandrasekaran M. (2015). Red pigment production by *Monascus purpureus* using sweet potato-based medium in submerged fermentation. Nutrafoods.

[201] Silbir S., Goksungur Y. (2019). Natural red pigment production by *Monascus purpureus* in submerged fermentation systems using a food industry waste: Brewer’s spent grain. Foods.

[202] Martin A.M., Lu C., Patel T.R. (1993). Growth parameters for the yeast *Rhodotorula rubra* grown in peat extracts. J. Ferment. Bioeng..

[203] Meyer P.S., du Preez J.C. (1994). Astaxanthin production by a *Phaffia rhodozyma* mutant on grape juice. World J. Microbiol. Biotechnol..

[204] Buzzini P., Martini A. (1999). Production of carotenoids by strains of *Rhodotorula glutinis* cultured in raw materials of agro-industrial origin. Bioresour. Technol..

[205] An G., Jang B., Cho M. (2001). Cultivation of the carotenoidhyperproducing mutant 2A2 N of the red yeast *Xanthophyllomyces dendrorhous (Phaffia rhodozyma)* with molasses. J. Biosci. Bioeng..

[206] Bhosale P., Gadre R.V. (2001). β-carotene production in sugarcane molasses by a *Rhodotorula glutinis* mutant. J. Ind. Microbiol. Biotechnol..

[207] Libkind D., Van Broock M. (2006). Biomass and carotenoid pigment production by patagonian native yeasts. World J. Microbiol. Biotechnol..

[208] Squina F.M., Yamashita F., Pereira J.L., Mercadante A.Z. (2002). Production of carotenoids by *Rhodotorula rubra* and *R. glutinis* in culture medium supplemented with sugar cane juice. Food Biotechnol..

[209] Buzzini P. (2001). Batch and fed-batch carotenoid production by Rhodotorula glutinis—Debaryomyces castellii co-cultures in corn syrup. J. Appl. Microbiol..

[210] Domínguez-Bocanegra A.R., Torres-Muñoz J.A. (2004). Astaxanthin hyperproduction by *Phaffia rhodozyma* (now *Xanthophyllomyces dendrorhous*) with raw coconut milk as sole source of energy. Appl. Microbiol. Biotechnol..

[211] Teo I.T.N., Chui C.H., Tang J.C.O., Lau F.Y., Cheng G.Y.M., Wong R.S.M., Kok S.H.L., Cheng C.H., Chan A.S.C., Ho K.P. (2005). Antiproliferation and induction of cell death of *Phaffia rhodozyma (Xanthophyllomyces dendrorhous)* extract fermented by brewer malt waste on breast cancer cells. Int. J. Mol. Med..

[212] Zheng Y.G., Hu Z.C., Wang Z., Shen Y.C. (2006). Large-scale production of astaxanthin by *Xanthophyllomyces dendrorhous*. Food Bioprod. Process..

[213] Tinoi J., Rakariyatham N., Deming R.L. (2006). Utilization of mustard waste isolates for improved production of astaxanthin by *Xanthophyllomyces dendrorhous*. J. Ind. Microbiol. Biotechnol..

[214] Chandi G.K., Singh S.P., Gill B.S., Sogi D.S., Singh P. (2010). Optimization of carotenoids by *Rhodotorula glutinis*. Food Sci. Biotechnol..

[215] Taskin M., Sisman T., Erdal S., Kurbanoglu E.B. (2011). Use of waste chicken feathers as peptone for production of carotenoids in submerged culture of *Rhodotorula glutinis* MT-5. Eur. Food Res. Technol..

[216] Kaur B., Chakraborty D., Kaur H. (2012). Production and stability analysis of yellowish pink pigments from *Rhodotorula rubra* MTCC 1446. Internet J. Microbiol..

[217] Bagy M.M.K., Abd-Alla M.H., Nafady N.A., Morsy F.M., Mahmoud G.A. (2016). Bioconversion of plant wastes to β-carotene by *Rhodotorula glutinis* KU550702. Eur. J. Biol. Res..

[218] Sharma R., Ghoshal G. (2020). Optimization of carotenoids production by *Rhodotorula mucilaginosa* (MTCC-1403) using agro-industrial waste in bioreactor: A statistical approach. Biotechnol. Rep..

[219] Elkenawy N.M., Yassin A.S., Elhifnawy H.N., Amin M.A. (2017). Optimization of prodigiosin production by *Serratia marcescens* using crude glycerol and enhancing production using gamma radiation. Biotechnol. Rep..

[220] Kalra R., Conlan X.A., Goel M. (2020). Fungi as a potential source of pigments: Harnessing filamentous fungi. Front. Chem..

[221] Kuo F.S., Chien Y.H., Chen C.J. (2012). Effects of light sources on growth and carotenoid content of photosynthetic bacteria *Rhodopseudomonas palustris*. Bioresour. Technol..

[222] Gmoser R., Ferreira J.A., Taherzadeh M.J., Lennartsson P.R. (2019). Post-treatment of fungal biomass to enhance pigment production. Appl. Biochem. Biotechnol..

[223] Palacio-Barrera A.M., Areiza D., Zapata P., Atehortúa L., Correa C., Peñuela-Vásquez M. (2019). Induction of pigment production through media composition, abiotic and biotic factors in two filamentous fungi. Biotechnol. Rep..

[224] Liu Z.Q., Zhang J.F., Zheng Y.G., Shen Y.C. (2008). Improvement of astaxanthin production by a newly isolated *Phaffia rhodozyma* mutant with low-energy ion beam implantation. J. Appl. Microbiol..

[225] Najafi N., Ahmadi A.R., Hosseini R., Golkhoo S. (2011). Gamma irradiation as a useful tool for the isolation of astaxanthin-overproducing mutant strains of *Phaffia rhodozyma*. Can. J. Microbiol..

[226] Yen H.W., Zhang Z. (2011). Enhancement of cell growth rate by light irradiation in the cultivation of *Rhodotorula glutinis*. Bioresour. Technol..

[227] De Carvalho J.C., Cardoso L.C., Ghiggi V., Woiciechowski A.L., De Souza Vandenberghe L.P., Soccol C.R., Brar S., Dhillon G., Soccol C. (2014). Microbial pigments. Biotransformation of Waste Biomass into High Value Biochemicals.

[228] Mata-Gómez L.C., Montañez J.C., Méndez-Zavala A., Aguilar C.N. (2014). Biotechnological production of carotenoids by yeasts: An overview. Microb. Cell Fact..

[229] Khatoon H., Kok Leong L., Abdu Rahman N., Mian S., Begum H., Banerjee S., Endut A. (2018). Effects of different light source and media on growth and production of phycobiliprotein from freshwater cyanobacteria. Bioresour. Technol..

[230] Walter A., de Carvalho J.C., Soccol V.T., de Faria A.B.B., Ghiggi V., Soccol C.R. (2011). Study of phycocyanin production from spirulina platensis under different light spectra. Brazilian Arch. Biol. Technol..

[231] Mishra S.K., Shrivastav A., Maurya R.R., Patidar S.K., Haldar S., Mishra S. (2012). Effect of light quality on the C-phycoerythrin production in marine cyanobacteria *Pseudanabaena* sp. isolated from Gujarat coast, India. Protein Expr. Purif..

[232] Sharma R., Sharma V.K. (2015). Effect of ultraviolet-B radiation on growth and pigments of *Chlorella vulgaris*. J. Indian Bot. Soc..

[233] Kamath B.S., Vidhyavathi R., Sarada R., Ravishankar G.A. (2008). Enhancement of carotenoids by mutation and stress induced carotenogenic genes in Haematococcus pluvialis mutants. Bioresour. Technol..

[234] Depauw F.A., Rogato A., D’Alcalá M.R., Falciatore A. (2012). Exploring the molecular basis of responses to light in marine diatoms. J. Exp. Bot..

[235] Yi Z., Xu M., Magnusdottir M., Zhang Y., Brynjolfsson S., Fu W., Martin-Jézéquel V. (2015). Photo-oxidative stress-driven mutagenesis and adaptive evolution on the marine diatom *Phaeodactylum tricornutum* for enhanced carotenoid accumulation. Mar. Drugs.

[236] Kobayashi M., Kakizono T., Nishio N., Nagai S. (1992). Effects of light intensity, light quality, and illumination cycle on astaxanthin formation in a green alga, *Haematococcus pluvialis*. J. Ferment. Bioeng..

[237] Xu Y., Harvey P.J. (2019). Carotenoid production by *Dunaliella salina* under red light. Antioxidants.

[238] Fu W., Guomundsson Ó., Paglia G., Herjólfsson G., Andrésson Ó.S., Palsson B.O., Brynjólfsson S. (2013). Enhancement of carotenoid biosynthesis in the green microalga *Dunaliella salina* with light-emitting diodes and adaptive laboratory evolution. Appl. Microbiol. Biotechnol..

[239] Fu W., Gudmundsson O., Feist A.M., Herjolfsson G., Brynjolfsson S., Palsson B. (2012). Maximizing biomass productivity and cell density of *Chlorella vulgaris* by using light-emitting diode-based photobioreactor. J. Biotechnol..

[240] Yi Z., Su Y., Xu M., Bergmann A., Ingthorsson S., Rolfsson O., Salehi-Ashtiani K., Brynjolfsson S., Fu W. (2018). Chemical mutagenesis and fluorescence-based high-throughput screening for enhanced accumulation of carotenoids in a model marine diatom *Phaeodactylum tricornutum*. Mar. Drugs.

[241] Rokem J.S., Weitzman P. (1987). Prodigiosin formation by *Serratia marcescens* in a chemostat. Enzym. Microb. Technol..

[242] Wang X., Tao J., Wei D., Shen Y., Tong W. (2004). Development of an adsorption procedure for the direct separation and purification of prodigiosin from culture broth. Biotechnol. Appl. Biochem..

[243] Xu F., Xia S., Yang Q. Strategy for obtaining inexpensive prodigiosin production by Serratia marcescens. Proceedings of the 3rd International Conference on Chemical, Biological and Environmental Engineering.

[244] Chen W.C., Yu W.J., Chang C.C., Chang J.S., Huang S.H., Chang C.H., Chen S.Y., Chien C.C., Yao C.L., Chen W.M. (2013). Enhancing production of prodigiosin from *Serratia marcescens* C3 by statistical experimental design and porous carrier addition strategy. Biochem. Eng. J..

[245] Song M.J., Bae J., Lee D.S., Kim C.H., Kim J.S., Kim S.W., Hong S.I. (2006). Purification and characterization of prodigiosin produced by integrated bioreactor from *Serratia* sp. KH95. J. Biosci. Bioeng..

[246] Juang R.S., Yeh C.L. (2014). Adsorptive recovery and purification of prodigiosin from methanol/water solutions of *Serratia marcescens* fermentation broth. Biotechnol. Bioprocess Eng..

[247] Bae J., Moon H., Oh K.K., Kim C.H., Sil Lee D., Kim S.W., Hong S.I. (2001). A novel bioreactor with an internal adsorbent for integrated fermentation and recovery of prodigiosin-like pigment produced from *Serratia* sp. KH-95. Biotechnol. Lett..

[248] Domröse A., Klein A.S., Hage-Hülsmann J., Thies S., Svensson V., Classen T., Pietruszka J., Jaeger K.E., Drepper T., Loeschcke A. (2015). Efficient recombinant production of prodigiosin in *Pseudomonas putida*. Front. Microbiol..

[249] Said F.M., Razali M.A.A. Effect of factors on the red pigment production in the stirred drum bioreactor: Fractional factorial design approach. Proceedings of the 2nd International Conference on Biosciences and Medical Engineering (ICBME2019): Towards Innovative Research and Cross-Disciplinary Collaborations.

[250] Setiyono E., Adhiwibawa M.A.S., Indrawati R., Prihastyanti M.N.U., Shioi Y., Brotosudarmo T.H.P. (2020). An Indonesian Marine Bacterium, Pseudoalteromonas rubra, Produces Antimicrobial Prodiginine Pigments. ACS Omega.

[251] Sagar B.S.V., Deepak B.S., Tejaswini G.S., Aparna Y., Sarada J. (2019). Evaluation of prodigiosin pigment for antimicrobial and insecticidal activities on selected bacterial pathogens & household pests. Int. J. Sci. Res. Biol. Sci..

[252] Mathlom G.S., Hayder N.H., Mahmood M.S. (2018). Synergistic effect of biosurfactant and prodigiosin produced by *Serratia marcescens* as antimicrobial agent. Curr. Res. Microbiol. Biotechnol..

[253] Ramesh C., Vinithkumar N.V., Kirubagaran R., Venil C.K., Dufosse L. (2020). Applications of prodigiosin extracted from marine red pigmented bacteria *Zooshikella* sp. and actinomycete *Streptomyces* sp.. Microorganisms.

[254] Genes C., Baquero E., Echeverri F., Maya J.D., Triana O. (2011). Mitochondrial dysfunction in *Trypanosoma cruzi*: The role of *Serratia marcescens* prodigiosin in the alternative treatment of Chagas disease. Parasites and Vectors.

[255] Canuto J.A., Lima D.B., de Menezes R.R.P.P.B., Batista A.H.M., Nogueira P.C.D.N., Silveira E.R., Grangeiro T.B., Nogueira N.A.P., Martins A.M.C. (2019). Antichagasic effect of violacein from *Chromobacterium violaceum*. J. Appl. Microbiol..

[256] Kanelli M., Mandic M., Kalakona M., Vasilakos S., Kekos D., Nikodinovic-Runic J., Topakas E. (2018). Microbial production of violacein and process optimization for dyeing polyamide fabrics with acquired antimicrobial properties. Front. Microbiol..

[257] Hui C.Y., Guo Y., Liu L., Zhang N.X., Gao C.X., Yang X.Q., Yi J. (2020). Genetic control of violacein biosynthesis to enable a pigment-based whole-cell lead biosensor. RSC Adv..

[258] Ballestriero F., Daim M., Penesyan A., Nappi J., Schleheck D., Bazzicalupo P., Di Schiavi E., Egan S. (2014). Antinematode activity of violacein and the role of the insulin/IGF-1 pathway in controlling violacein sensitivity in *Caenorhabditis elegans*. PLoS ONE.

[259] Kumar V., Thakur V., Ambika, Kumar S., Singh D. (2018). Bioplastic reservoir of diverse bacterial communities revealed along altitude gradient of Pangi-Chamba trans-Himalayan region. FEMS Microbiol. Lett..

[260] Dua A., Chauhan K., Pathak H. (2014). Biotransformation of indigo pigment by indigenously isolated *Pseudomonas* sp. HAV-1 and assessment of its antioxidant property. Biotechnol. Res. Int..

[261] Grossart H.-P., Thorwest M., Plitzko I., Brinkhoff T., Simon M., Zeeck A. (2009). Production of a Blue Pigment (Glaukothalin) by MarineRheinheimeraspp. Int. J. Microbiol..

[262] DeBritto S., Gajbar T.D., Satapute P., Sundaram L., Lakshmikantha R.Y., Jogaiah S., Ito S. (2020). ichi Isolation and characterization of nutrient dependent pyocyanin from *Pseudomonas aeruginosa* and its dye and agrochemical properties. Sci. Rep..

[263] Srilekha V., Krishna G., Seshasrinivas V., Singaracharya M.A. (2018). Evaluation of wound healing and anti-inflammatory activity of a marine yellow pigmented bacterium, *Micrococcus* sp.. Indian J. Geo-Marine Sci..

[264] Fariq A., Yasmin A., Jamil M. (2019). Production, characterization and antimicrobial activities of bio-pigments by *Aquisalibacillus elongatus* MB592, *Salinicoccus sesuvii* MB597, and *Halomonas aquamarina* MB598 isolated from Khewra Salt Range, Pakistan. Extremophiles.

[265] Venil C.K., Devi P.R., Dufossé L., Patra J.K., Fraceto F.L., Das G., Campos E.V.R. (2020). Synthesis of pigment-mediated nanoparticles and its pharmacological applications. Green nanoparticles synthesis and biomedical applications.

[266] Mapari S.A.S., Nielsen K.F., Larsen T.O., Frisvad J.C., Meyer A.S., Thrane U. (2005). Exploring fungal biodiversity for the production of water-soluble pigments as potential natural food colorants. Curr. Opin. Biotechnol..

[267] Nigam P.S., Luke J.S. (2016). Food additives: Production of microbial pigments and their antioxidant properties. Curr. Opin. Food Sci..

[268] Lagashetti A.C., Dufossé L., Singh S.K., Singh P.N. (2019). Fungal pigments and their prospects in different industries. Microorganisms.

[269] Duarte A.W.F., de Menezes G.C.A., e Silva T.R., Bicas J.L., Oliveira V.M., Rosa L.H., Rosa L.H. (2019). Antarctic fungi as producers of pigments. Fungi of Antarctica.

[270] Velmurugan P., Kamala-Kannan S., Balachandar V., Lakshmanaperumalsamy P., Chae J.C., Oh B.T. (2010). Natural pigment extraction from five filamentous fungi for industrial applications and dyeing of leather. Carbohydr. Polym..

[271] Venil C.K., Zakaria Z.A., Ahmad W.A. (2013). Bacterial pigments and their applications. Process Biochem..

[272] Agha Y.Y., Bahjat S.A., Thanoon M.F. (2019). Assessment of bacterial pigments as textile colorants. Indian J. Public Heal. Res. Dev..

[273] Yamashita K., Tokunaga E. (2020). Noninvasive and safe cell viability assay for *Paramecium* using natural pigment extracted from food. Sci. Rep..

[274] Yamashita K., Yamada K., Suzuki K., Tokunaga E. (2019). Noninvasive and safe cell viability assay for *Euglena gracilis* using natural food pigment. PeerJ.

[275] Yamashita K., Tagawa R., Higami Y., Tokunaga E. (2020). Noninvasive and safe cell viability assay for breast cancer MCF-7 cells using natural food pigment. Biology.

[276] Vila E., Hornero-Méndez D., Azziz G., Lareo C., Saravia V. (2019). Carotenoids from heterotrophic bacteria isolated from fildes Peninsula, King George Island, Antarctica. Biotechnol. Rep..

[277] Sajjad W., Din G., Rafiq M., Iqbal A., Khan S., Zada S., Ali B., Kang S. (2020). Pigment production by cold-adapted bacteria and fungi: Colorful tale of cryosphere with wide range applications. Extremophiles.

[278] Tapia C., López B., Astuya A., Becerra J., Gugliandolo C., Parra B., Martínez M. (2019). Antiproliferative activity of carotenoid pigments produced by extremophile bacteria. Nat. Prod. Res..

[279] Aki T., Hachida K., Yoshinaga M., Katai Y., Yamasaki T., Kawamoto S., Kakizono T., Maoka T., Shigeta S., Suzuki O. (2003). Thraustochytrid as a potential source of carotenoids. J. Am. Oil Chem. Soc..

[280] Park H., Kwak M., Seo J.W., Ju J.H., Heo S.Y., Park S.M., Hong W.K. (2018). Enhanced production of carotenoids using a Thraustochytrid microalgal strain containing high levels of docosahexaenoic acid-rich oil. Bioprocess Biosyst. Eng..

[281] Morocho-Jácome A.L., Ruscinc N., Martinez R.M., de Carvalho J.C.M., Santos de Almeida T., Rosado C., Costa J.G., Velasco M.V.R., Baby A.R. (2020). (Bio)Technological aspects of microalgae pigments for cosmetics. Appl. Microbiol. Biotechnol..

[282] Kannaujiya V.K., Kumar D., Pathak J., Sinha R.P., Mishra A.K., Tiwari D.N., Rai A.N. (2018). Phycobiliproteins and Their Commercial Significance. Cyanobacteria: From Basic Science to Applications.

[283] Sonani R.R. (2016). Recent advances in production, purification and applications of phycobiliproteins. World J. Biol. Chem..

[284] Sonani R.R., Rastogi R.P., Madamwar D., Rastogi R., Madamwar D., Pandey A. (2017). Natural antioxidants from algae: A therapeutic perspective. Algal Green Chemistry: Recent Progress in Biotechnology.

[285] Gastineau R., Turcotte F., Pouvreau J.B., Morançais M., Fleurence J., Windarto E., Prasetiya F.S., Arsad S., Jaouen P., Babin M. (2014). Marennine, promising blue pigments from a widespread *Haslea* diatom species complex. Mar. Drugs.

[286] Yoshida A., Sasaki H., Toyama T., Araki M., Fujioka J., Tsukiyama K., Hamada N., Yoshino F. (2017). Antimicrobial effect of blue light using *Porphyromonas gingivalis* pigment. Sci. Rep..

[287] Leelanarat K., Katsuta Y., Katsuragi H., Watanabe F. (2020). Antibacterial activity of blue high-power light-emitting diode-activated flavin mononucleotide against *Staphylococcus aureus* biofilm on a sandblasted and etched surface. Photodiagnosis Photodyn. Ther..

[288] Food and Nutrition Board (1971). FNB Food Colors.

[289] Carvalho J.C., Pandey A., Babitha S., Soccol C.R. (2003). Production of *Monascus* biopigments: An overview. Agro Food Ind. Hi-Tech.

[290] Dufossé L., Galaup P., Yaron A., Arad S.M., Blanc P., Murthy K.N.C., Ravishankar G.A. (2005). Microorganisms and microalgae as sources of pigments for food use: A scientific oddity or an industrial reality?. Trends Food Sci. Technol..

[291] Venil C.K., Aruldass C.A., Dufossé L., Zakaria Z.A., Ahmad W.A. (2014). Current perspective on bacterial pigments: Emerging sustainable compounds with coloring and biological properties for the industry-an incisive evaluation. RSC Adv..

[292] Saini R.K., Keum Y.S. (2017). Progress in Microbial Carotenoids Production. Indian J. Microbiol..

[293] McWilliams A. The Global Market for Carotenoids. https://www.bccresearch.com/market-research/food-and-beverage/the-global-market-for-carotenoids.html.

[294] Deinove Carotenoids Market Natural Carotenoids Meeting Consumer Needs. http://www.deinove.com/en/profile/strategy-and-markets/carotenoids-market/.

[295] Bhosale P., Bernstein P.S. (2005). Microbial xanthophylls. Appl. Microbiol. Biotechnol..

[296] Lin J.H., Lee D.J., Chang J.S. (2015). Lutein production from biomass: Marigold flowers versus microalgae. Bioresour. Technol..

[297] Markets M. Carotenoids Market by Type (Astaxanthin, Beta-Carotene, Lutein, Lycopene, Canthaxanthin, and Zeaxanthin), Application (Feed, Food & Beverages, Dietary Supplements, Cosmetics, and Pharmaceuticals), Source, Formulation, and Region—Global Forecast to 2026. https://www.marketsandmarkets.com/Market-Reports/carotenoid-market-158421566.html.

[298] Grand View Research Dyes and Pigments Market Size, Share & Trends Analysis Report by Product (Dyes (Reactive, Vat, Acid, Direct, Disperse), Pigment (Organic, Inorganic)), By Application, By Region, And Segment Forecasts, 2020–2027. https://www.grandviewresearch.com/industry-analysis/dyes-and-pigments-market.

[299] Saini R.K., Keum Y.S. (2019). Microbial platforms to produce commercially vital carotenoids at industrial scale: An updated review of critical issues. J. Ind. Microbiol. Biotechnol..

[300] Aberoumand A. (2011). A Review Article on Edible Pigments Properties and Sources as Natural Biocolorants in Foodstuff and Food Industry. World J. Dairy Food Sci..

[301] Harris R.M. (1999). Coloring Technology for Plastics.

